# PLGA-Based Micro/Nanoparticles: An Overview of Their Applications in Respiratory Diseases

**DOI:** 10.3390/ijms24054333

**Published:** 2023-02-22

**Authors:** Xiaoping Guo, Xu Zuo, Zhengjie Zhou, Yinuo Gu, Haoyu Zheng, Xinlei Wang, Guoqiang Wang, Caina Xu, Fang Wang

**Affiliations:** 1 Department of Pathogeny Biology, College of Basic Medical Sciences, Jilin University, Changchun 130021, China; 2 Department of Biochemistry, College of Basic Medical Sciences, Jilin University, Changchun 130021, China; 3 Key Laboratory of Pathobiology, Ministry of Education, Jilin University, Changchun 130021, China

**Keywords:** PLGA micro-/nanoparticles, treatment of respiratory diseases, asthma, chronic obstructive pulmonary disease (COPD), drug delivery

## Abstract

Respiratory diseases, such as asthma and chronic obstructive pulmonary disease (COPD), are critical areas of medical research, as millions of people are affected worldwide. In fact, more than 9 million deaths worldwide were associated with respiratory diseases in 2016, equivalent to 15% of global deaths, and the prevalence is increasing every year as the population ages. Due to inadequate treatment options, the treatments for many respiratory diseases are limited to relieving symptoms rather than curing the disease. Therefore, new therapeutic strategies for respiratory diseases are urgently needed. Poly (lactic-co-glycolic acid) micro/nanoparticles (PLGA M/NPs) have good biocompatibility, biodegradability and unique physical and chemical properties, making them one of the most popular and effective drug delivery polymers. In this review, we summarized the synthesis and modification methods of PLGA M/NPs and their applications in the treatment of respiratory diseases (asthma, COPD, cystic fibrosis (CF), etc.) and also discussed the research progress and current research status of PLGA M/NPs in respiratory diseases. It was concluded that PLGA M/NPs are the promising drug delivery vehicles for the treatment of respiratory diseases due to their advantages of low toxicity, high bioavailability, high drug loading capacity, plasticity and modifiability. And at the end, we presented an outlook on future research directions, aiming to provide some new ideas for future research directions and hopefully to promote their widespread application in clinical treatment.

## 1. Introduction

Respiratory diseases are common and frequent diseases, and the main lesions are in the trachea, bronchi, lungs and chest. Patients with mild cases often manifest as cough, chest pain and affected breathing, while patients with severe cases have difficulty breathing, a sense of oxygen deprivation, and even respiratory failure and eventually death [1]. Respiratory diseases have always been a major disease plaguing human beings, especially since the outbreak of COVID-19 in 2019; the number of patients with respiratory diseases has been increasing day by day, seriously threatening human life and property security [2]. In 2016, more than 9 million deaths were associated with respiratory diseases, equivalent to 15% of global deaths. Among them, chronic obstructive pulmonary disease (COPD), a relatively common respiratory disease, ranks third among the top ten causes of death worldwide, causing 3.2 million deaths annually, accounting for 81.7% of the total number of deaths from chronic respiratory disease [3]. Asthma, another common respiratory disease, is known to be the most common chronic disease of childhood, and its prevalence has been increasing over the past three decades [4]. Currently, more than 300 million people worldwide suffer from asthma, and the number of patients suffering from asthma may increase to 400 million by 2025 [5,6].

Currently, drugs used to treat respiratory diseases are mainly administered orally, intravenously or inhaled by nebulization into the body for therapeutic purposes [7]. Among them, oral administration is the most preferred route of administration for most drugs due to the advantages of high patient compliance, low cost, ease of administration, non-invasiveness and safety [8]. However, the absorption of orally administered drugs is a complex process, and factors such as the physicochemical properties of the drug, the nature of the dosage form and the physiology of the gastrointestinal tract (GIT) can all affect drug absorption [9]. As there are several absorption barriers in the GIT that prevent foreign substances from entering the body, only certain specific types of molecules can pass through GIT [8,10].

Compared to oral administration, intravenous administration for respiratory diseases can avoid the penetration of the mucosal barrier. However, it is an invasive route of administration that may lead to poor patient compliance and high medical costs during long-term treatment [11]. Moreover, both the intravenous and enteral routes are exposed to partial in vivo clearance mechanisms (e.g., mononuclear phagocytes in the liver and spleen or first chemical modifications in the liver) on the filtration or metabolism of the active ingredient of the drug and on the non-selective distribution of the drug, resulting in lower plasma concentrations of the drug and reduced distribution of the drug at the site of the lesion, directly affecting the therapeutic effect of the drug and increasing the toxic effects on normal tissues [12].

Nebulized inhalation drug delivery can be an alternative to intravenous drug delivery to some extent, and it is a promising administration strategy. Nebulized inhalation treatment of respiratory diseases is the use of the gas flow principle; the water droplets will be impacted into a fine aerosol suspended in the gas through the respiratory tract so that the drug is delivered directly to the lesion so as to achieve the purpose of treatment, with the advantages of easy operation, fast effects and drug delivery directly to the lesion [13,14]. Biomolecules such as peptides, proteins, deoxyribonucleic acid (DNA) and ribonucleic acid (RNA) can be administered by nebulized inhalation, which can significantly increase the bioavailability of biomolecules compared to other drug delivery strategies [15]. The use of nebulized inhalation antibiotics for pulmonary infections can rapidly achieve effective concentrations at the lesion, avoid frequent oral or intravenous administration of high doses of antibiotics, thereby reducing systemic adverse effects, and has promising applications in the treatment of pulmonary bacterial infections and prevention of bacterial resistance [16]. However, nebulized inhalation drug delivery has some disadvantages. For example, alveolar macrophages rapidly remove some of the inhaled drug, resulting in a limited duration of action and a reduced concentration of bacterial inhibition [15,17]. Studies have found that the vast majority of patients with respiratory diseases have varying degrees of airway mucus layer thickening, making it difficult for conventional drugs to reach the lesion [18].

**Figure 1:**
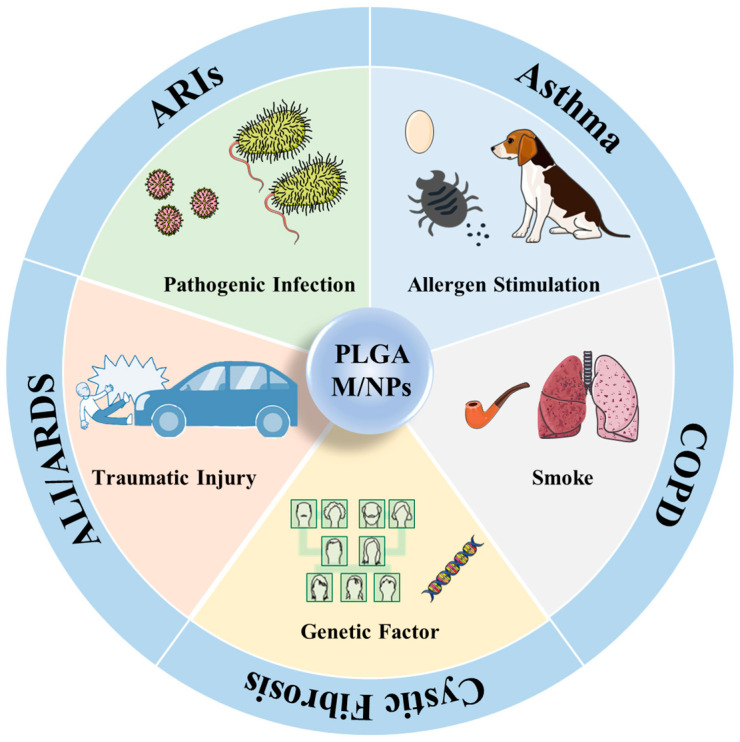
Application of PLGA M/NPs in different respiratory diseases.

The shortcomings of conventional drug delivery methods include low mucosal penetration efficiency, poor patient compliance with invasive drug delivery, high medical costs, the unsatisfactory biodistribution of drugs, toxic effects on normal tissues and so on. The disadvantages of conventional drug delivery methods include the influence of the internal environment on drug absorption, poor patient compliance with invasive drug delivery, high medical costs, the poor biodistribution of drugs, low mucosal penetration efficiency and toxic effects on normal tissues. However, most of the above-mentioned disadvantages of conventional drug delivery can be solved by micro- and nanotechnology. Micro- and nanotechnology have been widely developed in modern medicine and pharmacy applications, and micro/nanoparticles have significant advantages in achieving targeted drug delivery, drug sustained release, enhancing drug solubility, improving pharmacokinetics, prolonging blood circulation time and reducing the toxic side effects of drugs [19]. Due to their unique physicochemical properties, such as controllable size, good biocompatibility and low cytotoxicity, micro/nanoparticles have become a hot topic of research for researchers in many fields [20,21,22]. Among many micro/nanomaterials, poly (D,L-lactide-co-glycolide) (PLGA) copolymers are approved for medical use by the U.S. Food and Drug Administration (FDA) and the European Medicines Agency (EMA) as polymeric organic compounds with excellent biocompatibility, biodegradability and unique physical and chemical properties, making them one of the most popular and efficient polymers for drug delivery [23,24,25]. Compared with other conventional drug delivery strategies, PLGA M/NPs have many advantages, such as low toxicity, high bioavailability, controlled drug release, and direct action on lesions through multiple routes of drug delivery. However, their application in the treatment of respiratory diseases is rarely reported. Therefore, this review focused on the application and further prospects of PLGA M/NPs in respiratory diseases. In this review, we firstly introduced the synthesis and modification methods of PLGA M/NPs and summarized their progress in the treatment of respiratory diseases, including asthma, COPD, CF, acute lung injury (ALI)/acute respiratory distress syndrome (ARDS) and acute respiratory infections (ARIs) in the past few years. Common causes are as follows: allergen stimulation, such as animal hair, is a major factor in the development of asthma [26]; smoking is a major trigger for COPD [27]; CF is a genetic disorder usually caused by abnormal genes inherited from both parents [28]; intra- and extra-pulmonary factors such as blunt lung contusion are the main cause of ALI/ARDS [29]; and ARI is usually caused by pathogenic infections [30] (Figure 1). We hope that this review can stimulate scientific ideas to promote the early and widespread use of PLGA M/NPs in clinical applications for the treatment of respiratory diseases.

## 2. Synthesis Method of PLGA M/NPs

PLGA copolymers are widely used in drug delivery systems for the encapsulation of both hydrophilic and hydrophobic drugs [25,31]. It has been used in cancer treatment, wound healing and antibacterial, antioxidant and anti-inflammatory applications [32,33,34,35,36]. The main products of PLGA decomposition are lactic acid and glycolic acid, which are easily metabolized and cleared by the body; thus, their safety is greatly guaranteed [37]. PLGA M/NPs are commonly prepared by emulsion solvent evaporation, precipitation, spray drying, coacervation and low-temperature spray extraction. The most commonly used methods are briefly described below.

### 2.1. Single and Double Emulsion

In recent years, several methods for the preparation of PLGA M/NPs have been reported in the literature, among which the most commonly used methods are single and double emulsions, which are capable of encapsulating a wide range of drugs with different solubility [38]. The single emulsion (oil-in-water or O/W) method is suitable for encapsulation hydrophobic drugs. In this method, PLGA copolymers and the drugs are dissolved in an organic phase with volatility (e.g., benzyl alcohol, dichloromethane, ethyl acetate, etc.), and the liquid is added drop by drop to an aqueous phase containing a surfactant (e.g., polyvinyl alcohol (PVA), sodium cholate, etc.) in the aqueous phase, and then the mixture is sonicated, followed by stirring and evaporation to remove the organic solvent, and the diffusion of the organic solvent, and the counter-diffusion of water in the emulsion droplets result in the formation of polymer nanoparticles [39]. When encapsulating hydrophilic drugs, the double emulsion method is used to dissolve the drug in the aqueous phase first and dissolve PLGA copolymers in the organic phase, and then add the aqueous phase to the organic phase and perform the ultrasonic treatment to form a primary water-in-oil (W/O) emulsion. Then the primary emulsion is added to the aqueous phase, dissolved with a surfactant, and then sonicated and stirred to volatilize the organic solvent so that the microparticles are formed by water-in-oil-in-water (W/O/W) emulsion [40].

The single emulsion method has shown good results in the treatment of neurological diseases [41]. Fernández et al. used rasagiline mesylate microencapsulated into PLGA microspheres by the single emulsion method in an animal model of Parkinson’s disease and found that it promoted neuroprotection during the disease process [42]. The double emulsion method is often used to load proteins, nucleic acids and antiviral drugs [43,44,45]. Azizi et al. applied this method to prepare spherical PLGA NPs loaded with chondroitinase ABC for application in the treatment of spinal cord injury, which had potential as drug candidates for spinal cord repair, functional recovery and axonal regeneration [46]. Both single and double emulsion methods can control the size of microparticles by varying the ratio of drugs to PLGA copolymers, PLGA copolymers concentration, organic solvent, surfactant concentration in the aqueous phase, the nature of the solvent and the stirring speed. However, both methods usually have batch-to-batch variation, and the stability of the carriers of protein drug carriers prepared by these methods is limited due to the degradation of the protein at the water interface and the huge pressure of homogenization leading to the unfolding of the protein sheets [47].

### 2.2. Spray Drying Method

The basic principle of the spray drying method is to use the atomizer to disperse the material liquid into fine droplets and evaporate the solvent rapidly in the hot drying medium to form dry powder products, generally including four stages: (1) Atomization of the material liquid; (2) Contact mixing of the fog group with the hot drying medium; (3) Evaporation drying of the fog droplets; (4) Separation of the dry product from the drying medium. The spray drying method has the advantages of speed, convenience and few processing parameters and is suitable for industrially scalable processing [48,49]. This method requires the preparation of water in oil or solid in oil emulsions, which is then sprayed in a hot air stream to solidify the microparticles. The solvent used in the emulsion depends on the hydrophilic and hydrophobic nature of the encapsulated drug [40]. The spray drying method is suitable for wrapping hydrophilic and hydrophobic drugs, and due to the mild preparation conditions, it can also be used to wrap some condition-sensitive compounds. It has the advantages of speed, convenience and few processing parameters, making it suitable for industrial scale-up production. The main disadvantage of this method is that some of the emulsion will adhere to the inside of the nano-sprayer and cannot be recovered, leading to waste of the product [38,50,51,52]. In recent years, the olfactory mucosa has been recognized as a pathway to the brain and central nervous system, causing it to circumvent restrictions such as the blood-brain barrier [53]. Lena et al. used the spray drying method to develop homogeneous and reproducible PLGA NPs with high encapsulation rates and smooth surfaces and embedded them in chitosan particles, thus enhancing their absorption into the olfactory mucosa [54]. In a clinical study, poly (methacrylic acid-co-methyl methacrylate) (Eudragit-S100) encapsulated PLGA NPs prepared by spray drying method were demonstrated to have high oral bioavailability, enhanced sustained release and good targeting [55].

### 2.3. Precipitation Method

The precipitation method is a simple step, low cost, and the ability to build structure and function on demand synthesis technique [56]. Precipitation differs from emulsion-based methods (emulsification-diffusion, emulsion-evaporation and salting-out techniques) in that it is a rapid and straightforward method for the synthesis of micro/nanoparticles. In this method, PLGA copolymers and drugs are first dissolved into a polar organic solvent and then added to a large amount of aqueous phase, and the whole system undergoes phase separation, leading to the formation of PLGA M/NPs encapsulated with the drug, and then the organic solvent can be simply removed by evaporation [57]. Unfortunately, this method is not suitable for wrapping hydrophilic drugs because hydrophilic drugs cannot form favorable interactions with PLGA copolymers in the aqueous phase [58]. Razan et al. successfully prepared paclitaxel-PLGA-NPs to inhibit the proliferation of MCF-7 breast cancer cells using the nano-precipitation technique. They found that paclitaxel-loaded PLGA-NPs exhibited high efficacy against MCF-7 cells while showing no toxicity to normal MCF-10A cells [59]. In addition, Arjun et al. investigated PLGA-based NPs containing folic acid-targeted genistein loading by nanoprecipitation and demonstrated that they significantly enhanced cellular uptake and thus exerted better anticancer activity [60].

### 2.4. Coacervation Method

Coacervation is a method for preparing nanoscale or micron-scale biodegradable polymers by liquid-liquid phase separation techniques. It can facilitate polymer interactions by changing the ionic strength within the system, changing the temperature or adding a non-solvent to precipitate the polymer [61]. Coacervation methods are divided into mono-coacervation and complex-coacervation methods. The mono-coacervation method uses a hydrophilic electrolyte or a non-electrolyte as a coagulant to reduce the solubility of nanomaterials and cause them to agglomerate into microparticles. The complex-coacervation method uses two polymer materials with opposite charges to form a vesicle, which reduces the solubility and causes the microparticles to agglomerate and precipitate out of the solvent [40,62]. Peter et al. used the coacervation method to produce flexible PLGA-based implants for loading ciprofloxacin hydrochloride and showed that ciprofloxacin hydrochloride could be released at a controlled rate in vitro for up to 65 days [63].

When using the single and double emulsion method of particle preparation, the choice of solvent and mixing rate can affect the encapsulation efficiency and final particle size, so the particles are often used as injectable microsphere formulations [40]. Due to the mild preparation conditions of the spray drying method, it is often used to encapsulate environmentally sensitive contents such as nucleic acids and proteins to protect them from significant loss of activity. However, the disadvantage is that the particles adhere to the inner wall of the spray dryer during the synthesis process, causing some degree of loss [50,64,65,66]. The precipitation method is the simplest laboratory-based method for the preparation of polymer particles that has been consistently reported to date [67]. This synthetic method allows the synthesis of amphiphilic particles and is prepared without the need for high-shear homogenization techniques, ultracentrifugation or surfactants. However, this method has significant disadvantages, such as the concentration of the resulting particles is usually very low, and the particles cannot be lyophilized using freeze-drying techniques [68]. Furthermore, the coacervation method is a process focused on the preparation of micron-scale biodegradable polymer encapsulation formulations through liquid-liquid phase separation techniques. The desired morphology and size of the microspheres can be obtained by adjusting processing parameters such as polymer concentration, quenching temperature, quenching time and solvent composition [69,70,71]. In summary, different synthetic methods allow easy processing and fabrication of PLGA particles of various shapes and sizes that can be adapted to deliver different drugs as well as different delivery methods. This reinforces the fact that PLGA polymer is a very promising drug delivery vehicle. The following is the summary of the above synthesis methods and their application (Table 1).

## 3. Modification of PLGA M/NPs

In order to make PLGA M/NPs well-targeted, long-circulating in vivo and readily available for cellular uptake, the particles are generally modified by cationic, hydrophilic and targeting modifications [93]. The fundamental purpose of employing M/NPs in drug delivery is to use these carriers to carry and protect the loaded drugs and ultimately deliver them to the intended site. In practice, however, the fate of PLGA M/NPs in vivo is influenced by many factors, such as their surface charge, hydrophilicity/hydrophobicity and in vivo targeting ability [25]. In order to synthesize PLGA M/NPs with the desired properties, their precise design is required. To this end, surface modification has become the main strategy for solving these problems. [93].

### 3.1. Cationic Modification

PLGA M/NPs are widely used as a carrier for drug delivery systems due to their excellent biocompatibility and biodegradability, but they cannot easily adhere to certain negatively charged mucous membranes in the body because of their inherent negative charge. The intestinal mucosa contains many mucins with sialic acid, which are negatively charged, and it is difficult for PLGA M/NPs to adhere to its surface due to mutual repulsion of the same charges [94]. Therefore, cationic modification of PLGA M/NPs can greatly improve their ability to adhere to certain sites in vivo. Commonly used cationic modification substances include cetyltrimethylammonium bromide (CATB), chitosan (CS) and so on. Ramovatar et al. tested the anticancer activity of PLGA NPs encapsulated with curcumin modified with CATB in a triple negative breast cancer cell line (MDA-MB-231 cells) and showed significantly better cellular uptake, cytotoxicity and anticancer activity than unmodified PLGA NPs [93]. In addition, Dilip et al. demonstrated that chitosan-modified PLGA NPs had clearance, were readily absorbed by the body and induced significant systemic and mucosal immune responses in nasal vaccine delivery [95].

### 3.2. Hydrophilic Modification

Polyethylene glycol (PEG) is the most commonly used hydrophilic modified copolymer and is now used in many applications because of its good biocompatibility, water dispersibility, stability and ease of grafting [96]. Vllasaliu et al. and Owens et al. demonstrated that after PEG modification, the hydrated layer of PEG chains could effectively prevent the recognition and binding of tonin proteins to plasma proteins and reduce the phagocytosis of the reticuloendothelial system (RES), thus increasing the stability of drug-loaded nanoparticles, prolonging the in vivo circulation time and achieving controlled drug release [97,98]. Besides, Zohreh et al. used low molecular weight chitosan (LMWC) as a surface coating encapsulated on the outside of PLGA NPs. The hydrophilic LMWC layer protected the nanodrug under neutral pH conditions, effectively avoiding the clearance of nanoparticles by J774A.1 macrophages [99].

### 3.3. Targeting Modification

In recent years, advanced drug delivery systems have evolved from basic drug loading to intelligent drug delivery systems, which now focus on two main aspects, including targeting and stimulus responsiveness. Targeted micro/nanoparticles are able to target specific organs, tumors or inflammatory sites with significantly better therapeutic effects than non-targeted systems, which can largely reduce adverse drug reactions and multidrug resistance problems [100,101]. Targeted micro/nanoparticles are generally coated with specific ligands such as antibodies, small molecule peptides and aptamers that bind specifically and with high affinity to the target on a specific cell surface. Yang et al. achieved the targeting ability to the site of inflammation by transplanting γ3 peptide on the surface of PLGA NPs, through which the γ3 peptide could specifically bind to intercellular adhesion molecule-1 (ICAM-1). The drug-loaded γ3 PLGA NPs exhibited excellent antibacterial ability and effectively reduced inflammation and immune response in mice with acute lung infection [102]. In addition, Zhang et al. found that PLGA MPs loaded with (2-[(Aminocarbonyl)amino]-5-(4-fluorophenyl)-3-thiophenecarboxamide (TPCA-1) and coated with anti-ICAM-1 antibody on the surface were able to target inflamed lungs by intravenous injection, thereby reducing lung inflammation and injury [103]. Besides, Spence et al. prepared PLGA NPs modified with the natural Siglec ligand, di(α2→8) N-acetylneuraminic acid (α2,8 NANA-NP) and demonstrated that the modified PLGA NPs blocked lipopolysaccharide-induced production of inflammatory cytokines [104].

## 4. Application of PLGA M/NPs in Respiratory Diseases Treatment

### 4.1. Application of PLGA M/NPs in Asthma

Asthma is a heterogeneous disease characterized by chronic airway inflammation and airway hyperresponsiveness. The main features include chronic airway inflammation, high airway reactivity to multiple stimuli, variable and reversible airflow limitation and a series of airway structural changes over the course of the disease, known as airway remodeling [105]. Clinical manifestations include recurrent episodes of wheezing, shortness of breath, chest tightness or coughing, often occurring or worsening at night and in the early hours of the morning, with most patients relieved on their own or with treatment [106]. The common drugs used to treat asthma are mainly bronchodilators and inhaled corticosteroids. However, long-term application of corticosteroids can cause a variety of serious side effects and increase the mortality rate of patients. Several studies have demonstrated that delivery using micro-nanocarriers is a promising strategy compared to conventional delivery methods due to their ability to target tissues, enhancing therapeutic efficacy while minimizing systemic side effects [12].

**Figure 2:**
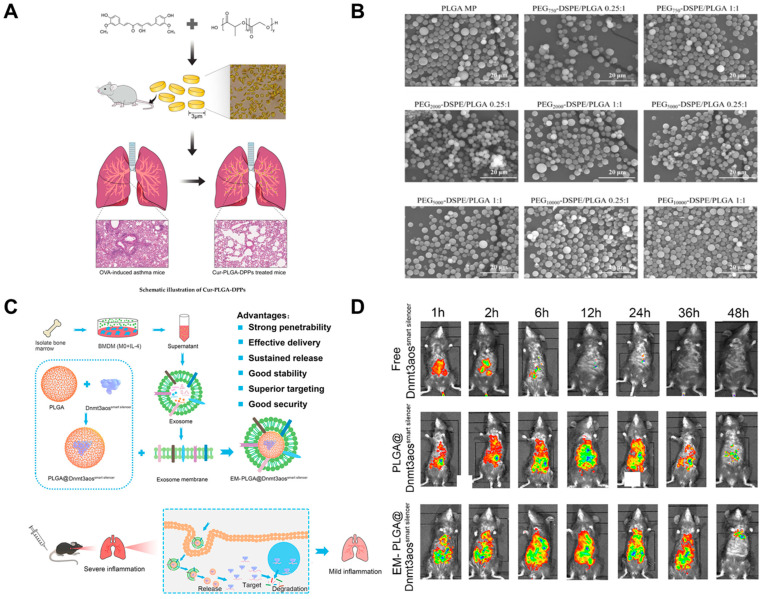
(**A**) Therapeutic efficacy of curcumin enhanced by microscale discoidal polymeric particles in a murine asthma model. Reproduced with permission from [110]. (**B**) Morphology of the budesonide-loaded PEG-DSPE-modified PLGA microspheres. Reproduced with permission from [113]. (**C**) Schematic illustration of exosome membrane-modified PLGA NPs deliver the Dnmt3aossmart silencer to ameliorate airway inflammation in allergic asthma. (**D**) In vivo tracking and tissue distribution of EM-PLGA@Dnmt3aossmart silencer in allergic asthma mice. Reproduced with permission from [119].

The morphology of the particles can affect their distribution in the organisms [107,108]. Decuzzi et al. found that the accumulation concentration of disc-shaped particles in the lung was significantly higher than that of spherical or quasi-hemispherical particles [109]. Park et al. prepared curcumin-containing PLGA-based microscale discoidal polymeric particles (Cur-PLGA-DPPs) and found that the number of inflammatory cells in asthmatic mice was significantly reduced by intravenous injection of Cur-PLGA-DPPs and the thickness of bronchial walls and the proliferation of cupped cells in asthmatic mice (Figure 2A) [110]. This might be attributed to enhanced curcumin bioavailability upon administration of curcumin as discoidal polymeric particles. Besides, whether the particles are porous or not also affects the distribution in the body. Oh et al. prepared porous PLGA MPs using ammonium bicarbonate as a porogenic agent and delivered PLGA MPs to mice via pulmonary administration. It was demonstrated that the lung absorption rate of porous PLGA MPs was significantly higher than that of non-porous PLGA MPs, and their therapeutic effects were also verified in asthmatic mice. Mice in the budesonide-loaded porous PLGA MPs group showed significantly lower levels of inflammation and significantly reduced airway hyperresponsiveness compared to the free budesonide and budesonide-loaded non-porous PLGA MPs-treated groups [88]. This might be because the lung uptake efficiency of porous PLGA particles is higher than that of non-porous PLGA particles. In addition, the size of the particles also affects how particles are deposited in the lungs. The study proved that large porous particles were more likely to accumulate in the lungs compared to small non-porous particles [111]. However, large nanoparticles have difficulty in crossing the mucus layer and maintaining good long circulation in vivo, whereas nanoparticles with a particle size of 200 nm or even smaller can easily pass through the mucus layer and maintain good long circulation in vivo [12].

Polyethylene glycol (PEG) modification is currently considered a strategy with the potential to penetrate the mucus layer of asthmatic lungs and reduce phagocytosis by macrophages. This is mainly because the hydrated layer of PEG chains effectively blocks the recognition of plasma proteins and myotonic proteins to inhibit their binding and reduces the phagocytosis of the reticuloendothelial system (RES), thus improving the stability of drug-loaded nanoparticles and enhancing the circulating half-life [97,98,112]. Li et al. investigated the effects of different PEG molecular weights and molar ratios on drug release, mucus penetration, macrophage uptake, lung accumulation and the in vivo distribution of PEG-modified PLGA MPs and showed that at a molar ratio of 1:1, PEG 2000 modified PLGA MPs could not only rapidly penetrate the mucus layer by pulmonary administration, but also accumulate in the lung for a long time (Figure 2B) [113].

With the development of treatment technology, some emerging treatments have also emerged, such as immunotherapy and gene therapy in the treatment of respiratory diseases. Allergen-specific immunotherapy (SIT) is currently an excellent treatment for allergic diseases. SIT can stimulate the increase of Treg cells in patients, suppress the production of specific IgE and inhibit the activity of allergen-specific effector T cells [114,115]. The tumor necrosis factor alpha-induced protein 3 (TNFAIP3/A20) has been shown to regulate a variety of immune cell functions and is involved in maintaining immune homeostasis in vivo [116]. Luo et al. synthesized the PLGA-based nanovaccine by wrapping ovalbumin (OVA) and A20 and applied this nanovaccine to a mouse model of asthma via nasal drops. The results showed that the as-prepared nanovaccine significantly inhibited the Th2 inflammatory response and promoted the production of Treg cells [117]. In addition, it has been demonstrated that thousands of long-stranded non-coding RNAs are differentially expressed in macrophage polarisation, with DNA methyltransferase 3A opposite strand (Dnmt3aos), located on the antisense strand of Dnmt3a, being a known lncRNA that plays a key role in macrophage polarisation and may therefore be a potential target for the treatment of allergic asthma [118]. Pei et al. wrapped a Dnmt3aossmart silencer consisting of three small interfering RNAs (siRNAs) and three antisense oligonucleotides (ASOs) inside PLGA and wrapped exosomal membranes from M2 macrophages around the overall surface of PLGA, exploiting the homing properties of the exosomal membranes for stable and efficient target delivery by intravenous injection (Figure 2C,D). The results suggested that by silencing Dnmt3aos, a key target gene in allergic asthma, airway inflammation could be reduced, leading to effective treatment of allergic asthma [119].

### 4.2. Application of PLGA M/NPs in COPD

COPD was ranked as the third leading cause of death globally in 2016, and with approximately 300 million people worldwide reported to have COPD as early as 2017, it has now become a major public health problem characterized by persistent respiratory symptoms and airflow limitation, usually associated with airway and/or alveolar abnormalities caused by significant exposure to harmful particles or gases [120]. Currently commonly used therapeutic drugs include bronchodilators (such as β1 adrenergic agonists, anticholinergics and theophyllines), glucocorticoids and expectorants, etc. [121]. However, the therapeutic effects of these drugs on COPD need to be improved, and their use is limited to the clinical treatment of COPD, and some of them have certain adverse effects; therefore, there is an urgent to develop safe and efficient drugs for the clinical treatment of COPD [122].

In the past few decades, it has been demonstrated that macrophages could be activated in different ways, and in addition to the classical activation of pro-inflammatory activation leading to host defense responses, macrophages activated in different ways could instead promote the dissipation of inflammation and facilitate tissue repair [123,124,125]. Therefore, the activation of macrophages through specific pathways to promote inflammation regression may have potential clinical applications. Noort et al. demonstrated that the small heat-shock protein alpha B-crystallin (HSPB5) could exert anti-inflammatory effects by activating macrophages via endosomal/phagosomal CD14 and Toll-like receptors 1 and 2. Subsequently, HSPB5 was encapsulated in porous PLGA MPs for the treatment of COPD, and it was found that HSPB5-loaded PLGA MPs were selectively taken up by alveolar macrophages during intratracheal administration, and the anti-inflammatory effect was associated with activation of an immune-regulatory macrophage response via TLR1/2, and CD14 as an essential co-receptor, which significantly inhibited neutrophil and lymphocyte infiltration in the lungs. In contrast, a 30-fold higher dose of free soluble HSPB5 still had no significant therapeutic effect [126].

**Figure 3:**
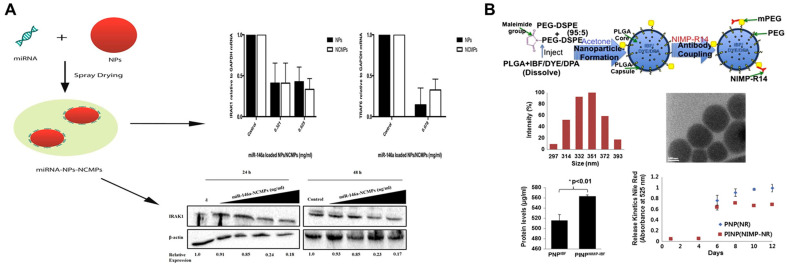
(**A**) Pulmonary delivery of nanocomposite microparticles (NCMPs) incorporating miR-146a for treatment of COPD. Reproduced with permission from [134]. (**B**) Synthesis, characterization, and kinetics of neutrophil-targeted and drug/dye-loaded nanoparticles (PINPNIMP-IBF). Reproduced with permission from [135].

RNA-based therapies have become a hot topic with the advent of RNA interference (RNAi) technology [127]. Among them, microRNAs (miRNAs) are widely used for the treatment of respiratory diseases because of their endogenous and editable nature to inhibit gene expression [128,129,130]. For example, miR-146a plays a key role in the pathogenesis of COPD, and it has potential therapeutic value due to its ability to downregulate the expression of interleukin-1 receptor-associated kinase (IRAK-1) and thus inhibit IL-1R signaling [131,132]. However, the precise delivery of miRNA to the desired site is considered to be one of the major problems in the development of miRNA therapies, and secondly, free miRNAs are unstable, and their physicochemical properties all affect the efficiency of these molecules in penetrating biological barriers [133]. Mohamed et al. used a mixture of L-leucine and mannitol as dispersion enhancers and protective excipients to prepare the nanocomposite microparticles (NCMPs) with cations, enabling them to adsorb and protect negatively charged miR-146a for efficient deposition in the deep lung and to downregulate IRAK-1 expression for anti-inflammatory effects via pulmonary administration, demonstrating their potential for treating COPD (Figure 3A) [134]. In addition, some small molecule drugs, such as ibuprofen, have been shown to treat COPD, but their low permeability across the mucosal barrier precludes their clinical use. To address this issue, Vij et al. loaded ibuprofen into PLGA NPs modified with a maleimide-capped PEG and PEG (5:95) copolymer. The aim was to reduce the neutrophil-mediated inflammatory response in COPD by exploiting the mucus inertness of PEG and the coupling properties of maleimide to achieve penetration and targeting (Figure 3B) [135]. The results showed that the NPs were able to specifically bind and release the drug into neutrophils after airway transport, thereby treating COPD by controlling neutrophil inflammation.

### 4.3. Application of PLGA M/NPs in CF

CF is an inherited disease caused by an abnormal gene inherited from both biological parents. CF affects nearly 70,000 patients worldwide and is most common, especially among non-Hispanic whites [136]. It is caused by mutations in the cystic fibrosis transmembrane conductance regulator (CFTR) gene resulting in decreased salt transport, decreased water movement, mucus accumulation, and obstruction of the airway, leading to recurrent infections triggering lung inflammation and injury. Respiratory failure is the most common cause of death in CF patients [137,138]. The current treatment for CF patients is mainly daily nebulized inhaled medications to dilute the mucus and help the patient expel it from the airways, along with oral or inhaled antibiotics to fight the infection. Lung transplantation is recommended for some patients with severe CF [139].

**Figure 4:**
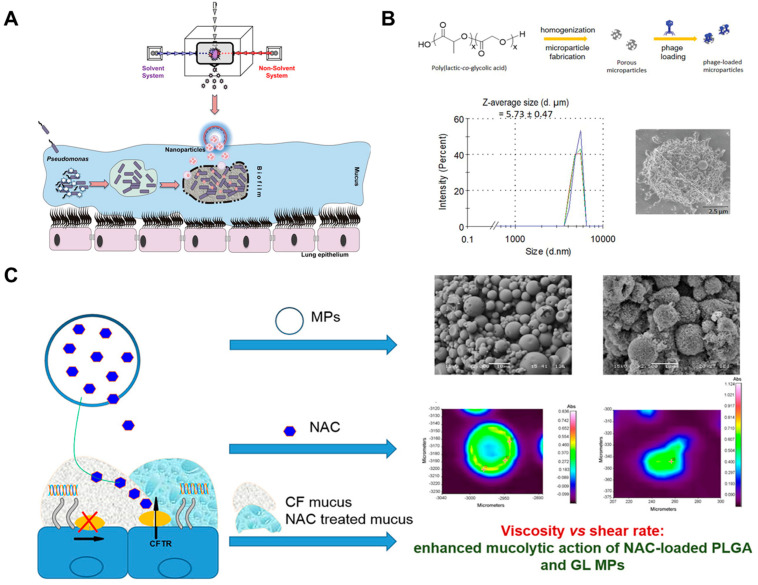
(**A**) Ciprofloxacin−loaded PLGA NPs against CF *P. aeruginosa* lung infections. Reproduced with permission from [144]. (**B**) Schematic diagram and characterization of fabrication and phage loading of porous PLGA MPs. Reproduced with permission from [151]. (**C**) Schematic illustration of a study of biodegradable microparticles that effectively reach and act on the mucus barrier of cystic fibrosis. Reproduced with permission from [158].

Chronic lung infections caused by a number of bacteria are the leading cause of death in CF patients, of which *Pseudomonas aeruginosa* has been shown to be the main pathogen [140]. Since *P. aeruginosa* infection can form bacterial biofilms that protect the bacteria from reaching minimum inhibition concentration (MIC) at the site of action and kill the bacteria, which leads to the emergence of bacterial drug resistance [141]. Therefore, achieving the controlled release of antibiotics at the lesion and increasing the penetration of the drug into the bacterial biofilm may be an effective strategy for treating infections with drug-resistant strains of bacteria [142,143]. The ciprofloxacin-loaded PLGA NPs were synthesized with a size of 190.4 ± 28.6 nm, with a drug encapsulation rate of 79%, and the nanoparticles were shown to be safe and non-toxic at the minimum inhibitory concentration (MIC). Overall, in the context of pulmonary drug delivery, they were expected to be effective in penetrating the mucus layer and crossing bacterial biofilms to exert antibacterial effects (Figure 4A) [144]. Exotoxin A (ETA) is the most virulent extracellular component of *P. aeruginosa* and can be extracted from over 90% of *P. aeruginosa* clinical isolates [145]. ETA is a T-dependent antigen that stimulates T cells and leads to the production of memory cells [146]. A novel PLGA-based vaccine candidate containing ETA (ETA-PLGA NPs) against *Pseudomonas aeruginosa* was synthesized and characterized in vitro and in vivo. The results showed that ETA could act as a suitable immunogenic substance to stimulate the immune response. Compared to the free ETA-treated group, ETA-PLGA NPs by intramuscular injection promoted the expression of cytokines such as TNF-α, INF-γ, IL-17A and IL-4 and enhanced the IgG response in immunized mice, indicating that ETA-PLGA NPs could increase its functional activity by reducing bacterial transmission [147].

Because of the ability of phages to kill bacteria within bacterial biofilms, their ability to suppress infections caused by drug-resistant strains, and their specificity for specific pathogens, phage therapy has been developed in recent years to treat drug-resistant bacteria as an alternative to antibiotic therapy [148,149]. In addition to *P. aeruginosa*, *Staphylococcus aureus* is also a common pathogenic bacterium that can cause pulmonary infections [150]. Kalelkar et al. designed PLGA MPs for the endotracheal delivery of phages active against *S. aureus* (Figure 4B). Phage loading onto the surface of MPs was effectively achieved by incubating MPS in a phage solution. In vitro, the MPs were able to infect and lyse *S. aureus* and were also observed to inhibit the growth of *S. aureus* in the supernatant of mucoid sputum and sputum specimens from CF patients. The MPs also showed effective inhibition of *S. aureus* in a mouse model of acute lung infection [151].

Since the airway is the primary target site for CF treatment, inhaled drug delivery has become the primary treatment for CF [149,152]. However, the airways of patients with severe respiratory disease are often covered by the mucus layer, a complex barrier composed of highly cross-linked mucin chains, water and other gel-like components that can prevent the accumulation of various drugs in the lungs and interfere with their efficacy [153,154,155,156]. N-acetyl cysteine (NAC) is a commonly used mucolytic agent that cleaves the disulfide bonds of mucin fibers and thus acts to reduce the viscosity and enlarge the pore size of mucus/sputum in vitro and in vivo [157]. Cristallini et al. synthesized PLGA MPs or gellan gum, and NAC was encapsulated into PLGA MPs to achieve the mucolytic effects (Figure 4C). The results showed that NAC could be released from the PLGA MPs and diffuse into the mucus, thereby achieving dilution of the mucus layer. Therefore, the authors concluded that the as-prepared PLGA MPs had potential as effective carriers for the treatment of CF [158].

### 4.4. Application of PLGA M/NPs in ALI/ARDS

ARDS refers to acute diffuse lung injury and subsequent development of acute respiratory failure caused by various intra- and extra-pulmonary pathogenic factors and is a common and severe clinical syndrome with high incidence and mortality [159,160]. Blunt chest trauma, pneumonia, inhalation injuries and ventilator-induced lung injury (VILI) have been reported as the main or pulmonary causes of ARDS [29]. The main pathological features are damage to the pulmonary microvascular endothelium and alveolar epithelium due to inflammation and increased vascular permeability, which in turn leads to pulmonary edema and hyaline membrane formation. The main pathophysiological changes are reduced lung volumes, reduced lung compliance and severe ventilation/blood flow ratio imbalance [161,162]. ALI and ARDS are two stages of the same disease process, with ALI representing the early and less severe stage and ARDS representing the later and more severe stage.

**Figure 5:**
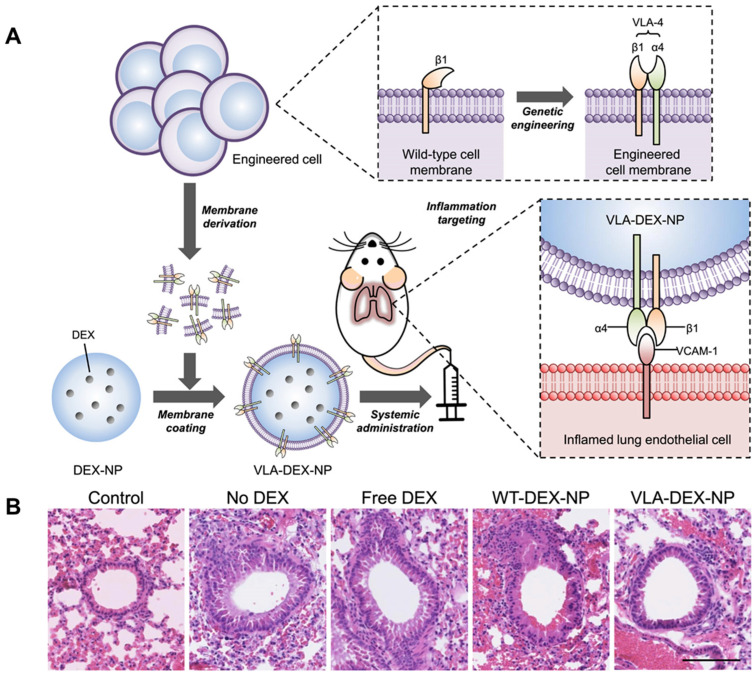
(**A**) Schematic illustration of genetically engineered cell membrane-coated nanoparticles for targeted drug delivery to inflamed lungs. (**B**) Mice were challenged with bacterial lipopolysaccharide and then treated with intravenous administration of VLA-DEX-NP in H&E lung histological sections (scale bar, 100 μm). Reproduced with permission from [166].

There is no specific effective therapy for ALI/ARDS, and early administration of dexamethasone can greatly reduce the duration of mechanical ventilation and mortality in patients with ARDS [163]. However, there are significant side effects associated with the long-term use of dexamethasone [164], and there is a need to develop delivery systems with lung-targeting potential. Kotta et al. synthesized dexamethasone-loaded lipopolymer PLGA MPs with pulmonary targeting using the single emulsion technique, administered intravenously to rats with lipopolysaccharide-induced ARDS model and were able to significantly inhibit the inflammatory response and reduce lung tissue damage. Therefore, this delivery system would contribute to the reduction of dose and frequency of drug administration, thereby reducing side effects [72]. Inflamed endothelial cells have been shown to upregulate vascular cell adhesion molecule-1 (VCAM-1) expression as a means of recruiting immune cells, such as leukocytes expressing very late antigen-4 (VLA-4) [165]. Park et al. used wild-type cells genetically engineered to express VLA-4 consisting of integrins α4 and β1, which were then wrapped in dexamethasone-loaded PLGA NPs through the plasma membrane of the genetically engineered cells, resulting in the synthesis of a cell membrane-encapsulated DEX-NP expressing VLA-4 (VLA-DEX-NP), which was administered intravenously to mice (Figure 5A) [166]. The study further confirmed the therapeutic effect of VLA-DEX-NP on lung inflammation through hematoxylin and eosin (H&E) stained lung sections (Figure 5B). Compared with the control group, free DEX group, and WT-DEX-NP group, the VLA-DEX-NP group showed the least inflammatory cell infiltration and no significant thickening of the bronchial wall, confirming the significant therapeutic effect of VLA-DEX-NP on lung inflammation.

In addition, oxidative stress has been reported to play a crucial role in the development and progression of ALI, and mitochondria are the main organelles attacked by reactive oxygen species (ROS). Therefore, the possibility of targeted delivery of antioxidants to mitochondria has become a difficult and hot topic in ALI research [167,168]. Jin et al. designed sialic acid (SA)-modified lung-targeted MPs, which encapsulated antioxidant-active curcumin inside, combined with E-selectin expressed on inflamed lung endothelial cells and increased PLGA MPs accumulation at the site of inflammation, targeted mitochondria for sustained release of curcumin [169]. The results showed that these MPs could increase the cellular uptake of inflammatory cells by binding to E-selectin receptors and inhibit apoptosis by reducing ROS production. In addition, the therapeutic effect of such MPs on murine models of ALI can be demonstrated in terms of both oxidative stress production and pro-inflammatory cytokine expression.

### 4.5. Application of PLGA M/NPs in ARIs

ARIs, also known as acute respiratory syndromes (ARSs), are responsible for about 4 million deaths annually and are the major cause of death among children under 5 years old [170,171]. Infections of the lower respiratory tract are mainly caused by bacteria and viruses, including conditions such as the common cold, bronchitis pharyngitis, laryngitis, tuberculosis and pneumonia [30].

**Figure 6:**
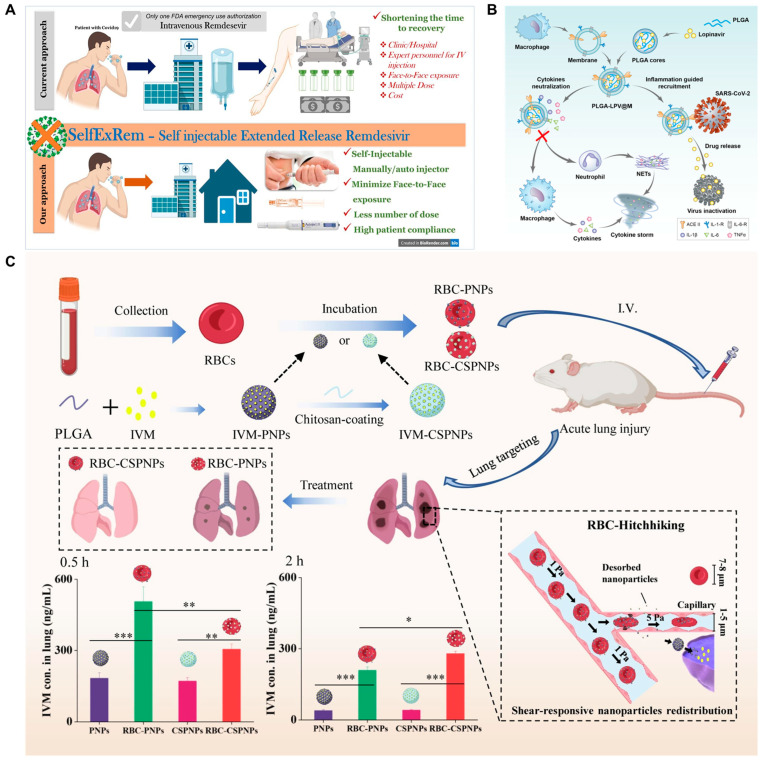
(**A**) Schematic representation of SelfExRem. Reproduced with permission from [180] (**B**) Schematic illustration of macrophage biomimetic nanocarriers based drug delivery system (PLGA-LPV@M) for anti-inflammation and targeted antiviral treatment in COVID-19. Reproduced with permission from [182]. (**C**) Diagram of the red blood cell (RBC)-hitchhiking strategy. Reproduced with permission from [184]. * *p* < 0.05, ** *p* < 0.01,*** *p* < 0.001.

With the emergence of new respiratory viruses and related diseases, such as the novel coronavirus SARS-CoV-2, which emerged in late 2019, and COVID-19 caused by its infection, the severity and extent of ARI are increasing each year [172,173]. COVID-19 mainly affects the lower respiratory system, and the most common symptoms in patients are fever, cough and shortness of breath. Other symptoms include malaise, vomiting, diarrhea, decreased sense of taste and smell, etc. In addition, vital organs such as the liver, heart, kidneys and gastrointestinal tract can be affected, leading to multi-organ complications [174,175]. In order to control the global spread of COVID-19, many preventive measures have been taken, such as home quarantine, home office and minimizing the movement of people. Unfortunately, even with these precautions, the cumulative number of confirmed cases per day in each country continues to rise, as there is no specific treatment or vaccine available for disease control [176,177]. Remdesivir (RDV) was one of the first drugs approved by the US Food and Drug Administration for the treatment of COVID-19, but its use has not been rationalized due to poor patient compliance with intravenous administration and the increased risk of transmission of infection from further interpersonal contact due to hospital infusions [178,179]. Patki et al. developed the self-injectable extended-release subcutaneous injection of RDV (SelfExRem) by using PLGA MPs, minimizing face-to-face contact, hospitalization and frequency of dosing, thereby significantly increasing the availability of RDV for patients in the early stages of infection and for those with limited hospital or clinical facilities (Figure 6A) [180].

Cytokine storm syndrome (CSS) is strongly associated with poor prognosis in COVID-19 [181]. Tan et al. developed an intravenous drug delivery system based on bionanocarriers for the anti-inflammatory and antiviral treatment of COVID-19 (Figure 6B). Lopinavir (LPV) was first encapsulated in PLGA NPs to form PLGA-LPV NPs, and then macrophage membranes were wrapped to form drug-loaded macrophage bionanocarriers (PLGA-LPV@M) due to the inherited surface receptors of macrophage membranes, PLGA@M could masquerade as mini-macrophages and competitively took up a variety of pro-inflammatory substances to inhibit the activation of macrophages and neutrophils, ultimately alleviating or stopping the progression of CSS so that they could significantly relieve inflammation and reduce tissue viral loading capacity. This study might have great application in the treatment of COVID-19 [182]. In addition, ivermectin (IVM) has been found to inhibit SARS-CoV-2 replication in vitro; however, the low plasma concentration of IVM in the lesion after oral administration makes oral-free IVM unable to achieve the desired therapeutic effect [183]. Zheng et al. synthesized PLGA NPs loaded with IVM, coated with chitosan (CS), and finally adsorbed on the surface of red blood cells (RBC) to prepare RBC-CSPNPs [184]. As the intravenously injected nanoparticles do not resist the shear stress between the RBC and the small capillaries, they are dislodged from the pulmonary capillaries [185], thus achieving the ability to target nanoparticles to the lung (Figure 6C). The study has shown that nanoparticles modified with CS significantly increase the adsorption efficiency of nanoparticles on RBC, resulting in enhanced targeting to the lung, prolonged circulation of nanoparticles and reduced clearance of nanoparticles in vivo.

*Staphylococcus aureus* has gradually become an important pathogen of pneumonia, and the emergence and infection of methicillin-resistant *Staphylococcus aureus* (MRSA) has aggravated the morbidity and mortality of patients [186,187]. Studies have shown that about 81% of patients with community-acquired pneumonia (CAP) caused by *S. aureus* infections become seriously ill, requiring intensive care therapy, with a mortality rate of 29% [188]. The production of virulence factors panton-valentine leucocidin (PVL), alpha-hemolysin (Hla) and biofilms are currently considered as several factors that may contribute to exacerbations of the disease [189,190]. Lin et al. designed lysozyme-encapsulated PLGA MPs for intravenous injection, which had a good affinity with the lung, high stability, effectively eliminated MRSA, reduced biofilm formation and had a good anti-inflammatory effect, thus improving the survival rate of mice [191]. In addition to *S. aureus*, *Pseudomonas aeruginosa* and *Klebsiella* are also the main pathogens of pneumonia [192,193]. Encapsulation of *P. aeruginosa* type III secretion system protein POPB and its chaperone protein pCRH in PLGA NPs could be used as nasal vaccination to protect mice from acutely fatal *P. aeruginosa* pneumonia [194]. In addition, Qu et al. synthesized PLGA MPs loaded with cefquinolone (CEQ) for the treatment of *Klebsiella* pneumonia. The results showed that intravenous administration of CEQ-loaded PLGA MPs had good lung accumulation and reduced lung bacterial colony counts and expression of inflammatory cytokines. Therefore, targeted delivery of CEQ was expected to be an alternative method for the control of important zoonotic pathogens [195].

## 5. Conclusions and Outlook

At present, the research and applications of micro- and nanotechnology in drug delivery are developing rapidly [196,197]. PLGA M/NPs are considered promising drug delivery vehicles because of their biocompatibility, high bioavailability, biodegradability, surface modifiability and active targeting [198,199]. Although the application of PLGA M/NPs offers more options for the prevention and treatment of respiratory diseases, there are still some shortcomings. For example, PLGA M/NPs have an impact on the stability of the loaded proteins during preparation and storage, mainly due to the accumulation of acidic monomers and lactic and glycolic acids generated by their hydrolysis within the delivery system, which leads to a significant decrease in the pH of the local microenvironment denaturing the encapsulated proteins [200]. And different methods of preparing PLGA M/NPs can also have different degrees of adverse effects on the secondary structure of certain proteins [201]. Besides, there may be potential safety issues with nanomaterials, and there are many studies showing that nanoparticles may have adverse effects on healthy tissues and organs [202,203]. What’s more, the lack of uniformity in particle size of nanomedicines resulting in poor reproducibility [204] and the high cost of preparation for commercialization and mass production are still many challenges [205].

In recent years, with the efforts of researchers, some solutions to the above problems have been found. To address the problems associated with protein degradation, loading, etc. There has been research into encapsulating proteins by complexing them with zinc or adding antacid excipients to the buffer to protect the protein activity [206]. To address potential safety issues, we can use means to accumulate as many M/NPs as possible in the diseased lungs. For example, positively charged micro-nanoparticles that do not effectively penetrate airway mucus can be modified with a cationic carrier on their surface to have a neutral or anionic group. In addition, active targeting could provide new ideas for optimizing drug delivery systems by exploring receptors that are expressed only at the lung site and modifying the surface of the M/NPs so that it can specifically bind to that receptor, thus enabling active targeting of the pathogenic site. Besides, M/NPs with responsive drug release capability can be designed to release drugs only at the site of disease, thereby improving therapeutic efficiency and reducing systemic toxicity [153,207,208,209,210,211].

Synthetic strategies for drug delivery based on micro- and nanotechnology have shown potential possibilities for the treatment of respiratory diseases at both the research and clinical levels. Currently, researchers have developed a number of PLGA M/NPs for the treatment of respiratory system diseases for in vitro experiments regarding pharmacology, biocompatibility and safety, followed by in vivo evaluation using corresponding animal models. To better utilize this technology, researchers should focus on understanding the mechanism of action of the drug or drug delivery system in vivo while taking into account the production methods to ensure a more rational design of M/NPs in the future. More importantly, we still have not established international standards for in vitro and in vivo studies of micro/nanomedicines, and the establishment of uniform standards will facilitate effective intercomparison of studies and thus promote the development of micro/nanomedicines. Development and challenges go hand in hand, and only the existence of currently unsolved problems can drive the development and depth of scientific research. In general, micro/nanomedicines are a technology with extremely long-term development value, and we believe that someday in the future, we will overcome these difficulties and realize the widespread application of micro/nanomedicines in clinical practice.

## Figures and Tables

**Table 1:** Summary of the synthesis methods and application.

Synthesis Method	Disease	Animal Model	Route of Administration	Ref.
Single and double emulsion	ALI/ARDS	Wistar rats	Intravenous injection	[72]
Single and double emulsion	Wound healing	Diabetic rats	Gavage administration	[73]
Single and double emulsion	Chronic glaucoma	Long Evans rats	Intracameral injection	[74]
Single and double emulsion	Breast cancer	Female nude mice	Hypodermic injection	[75]
Single and double emulsion	Myocardial infarction	Sprague–Dawley (SD) rats	Intravenous injection	[76]
Single and double emulsion	CF	C57BL/6 mice	Pulmonary administration	[77]
Single and double emulsion	CF	C57BL/6J female mice	Pulmonary administration	[78]
Spray drying	Pneumonia	Mice	Intravenous injection	[79]
Spray drying	Asthma	New Zealand rabbits	Intravenous injection	[80]
Spray drying	ALI/ARDS	Male FVB/NJ mice	Intramuscular injection	[81]
Spray drying	AIDS	Wistar rats	Gavage administration	[82]
Precipitation	Cancer	BALB/C mice	Intravenous injection	[83]
Precipitation	Glioblastoma	Wistar rats	Intravenous injection	[84]
Precipitation	Wound healing	Wistar rats	Gavage administration	[85]
Precipitation	Eye Inflammation	New Zealand rabbits	Ocular administration	[86]
Precipitation	Glioma	mice	Intravenous injection	[87]
Coacervation	Asthma	BALB/c mice	Pulmonary administration	[88]
Coacervation	Derzsy’s disease	Muscovy duck	Subcutaneous injection	[89]
Coacervation	Diphtheria	Guinea-pigs	Subcutaneous injection	[90]
Coacervation	Fungal infection	Mice	Intravenous injection	[91]
Coacervation	Cancer	Male Balb/c mice	Intravenous injection	[92]

## References

[1] Chan Y., Singh S.K., Gulati M., Wadhwa S., Prasher P., Kumar D., Kumar A.P., Gupta G., Kuppusamy G., Haghi M. (2022). Advances and applications of monoolein as a novel nanomaterial in mitigating chronic lung diseases. J. Drug Deliv. Sci. Technol..

[2] Hurgobin B., De Jong E., Bosco A. (2018). Insights into respiratory disease through bioinformatics. Respirology.

[3] Levine S.M., Marciniuk D.D. (2022). Global impact of respiratory disease: What can we do, together, to make a difference?. Chest.

[4] Poole J.A. (2014). Asthma is a major noncommunicable disease affecting over 230 million people worldwide and represents the most common chronic disease among children. Int. Immunopharmacol..

[5] Maciag M.C., Phipatanakul W. (2020). Prevention of asthma targets for intervention. Chest.

[6] Voskamp A., Kormelink T.G., van Wijk R.G., Hiemstra P., Taube C., de Jong E., Smits H.H. (2020). Modulating local airway immune responses to treat allergic asthma: Lessons from experimental models and human studies. Semin. Immunopathol..

[7] Patton J.S., Byron P.R. (2007). Inhaling medicines: Delivering drugs to the body through the lungs. Nat. Rev. Drug Discov..

[8] Ahadian S., Finbloom J.A., Mofidfar M., Diltemiz S.E., Nasrollahi F., Davoodi E., Hosseini V., Mylonaki I., Sangabathuni S., Montazerian H. (2020). Micro and nanoscale technologies in oral drug delivery. Adv. Drug Deliv. Rev..

[9] Abuhelwa A.Y., Williams D.B., Upton R.N., Foster D.J.R. (2017). Food, gastrointestinal pH, and models of oral drug absorption. Eur. J. Pharm. Biopharm..

[10] Amara S., Bourlieu C., Humbert L., Rainteau D., Carrière F. (2019). Variations in gastrointestinal lipases, pH and bile acid levels with food intake, age and diseases: Possible impact on oral lipid-based drug delivery systems. Adv. Drug Deliv. Rev..

[11] Luo C., Sun J., Du Y., He Z. (2014). Emerging integrated nanohybrid drug delivery systems to facilitate the intravenous-to-oral switch in cancer chemotherapy. J. Control Release.

[12] Zhong W., Zhang X., Zeng Y., Lin D., Wu J. (2021). Recent applications and strategies in nanotechnology for lung diseases. Nano Res..

[13] Sorino C., Negri S., Spanevello A., Visca D., Scichilone N. (2020). Inhalation therapy devices for the treatment of obstructive lung diseases: The history of inhalers towards the ideal inhaler. Eur. J. Intern. Med..

[14] Chandel A., Goyal A.K., Ghosh G., Rath G. (2019). Recent advances in aerosolised drug delivery. Biomed. Pharmacother..

[15] Patton J.S., Fishburn C.S., Weers J.G. (2004). The lungs as a portal of entry for systemic drug delivery. Proc. Am. Thorac. Soc..

[16] Bassetti M., Vena A., Russo A., Peghin M. (2020). Inhaled liposomal antimicrobial delivery in lung infections. Drugs.

[17] Sung J.C., Pulliam B.L., Edwards D.A. (2007). Nanoparticles for drug delivery to the lungs. Trends Biotechnol..

[18] Johnson D.C. (2011). Airway mucus function and dysfunction. N. Engl. J. Med..

[19] Mo R., Jiang T., Di J., Tai W., Gu Z. (2014). Emerging micro- and nanotechnology based synthetic approaches for insulin delivery. Chem. Soc. Rev..

[20] Bhattacharyya A., Janarthanan G., Kim T., Taheri S., Shin J., Kim J., Bae H.C., Han H.S., Noh I. (2022). Modulation of bioactive calcium phosphate micro/nanoparticle size and shape during in situ synthesis of photo-crosslinkable gelatin methacryloyl based nanocomposite hydrogels for 3D bioprinting and tissue engineering. Biomater. Res..

[21] Guo Z., Liu Y., Luo Y. (2022). Mechanisms of carotenoid intestinal absorption and the regulation of dietary lipids: Lipid transporter-mediated transintestinal epithelial pathways. Crit. Rev. Food Sci. Nutr..

[22] Meng H., Ye W., Wang C., Gao Z., Hu B., Wang C. (2022). Crystalline micro-nanoparticles enhance cross-linked hydrogels via a confined assembly of chitosan and gamma-cyclodextrin. Carbohydr. Polym..

[23] Swider E., Koshkina O., Tel J., Cruz L.J., de Vries I.J.M., Srinivas M. (2018). Customizing poly(lactic-co-glycolic acid) particles for biomedical applications. Acta Biomater..

[24] Essa D., Kondiah P.P.D., Choonara Y.E., Pillay V. (2020). The design of poly(lactide-co-glycolide) nanocarriers for medical applications. Front Bioeng. Biotechnol..

[25] El-Hammadi M.M., Arias J.L. (2022). Recent advances in the surface functionalization of PLGA-based nanomedicines. Nanomaterials.

[26] Papi A., Brightling C., Pedersen S.E., Reddel H.K. (2018). Asthma. Lancet.

[27] Mintz M., Barjaktarevic I., Mahler D.A., Make B., Skolnik N., Yawn B., Zeyzus-Johns B., Hanania N.A. (2023). Reducing the risk of mortality in chronic obstructive pulmonary disease with pharmacotherapy: A narrative review. Mayo Clin. Proc..

[28] Ehre C., Ridley C., Thornton D.J. (2014). Cystic fibrosis: An inherited disease affecting mucin-producing organs. Int. J. Biochem. Cell Biol..

[29] Lupu L., Palmer A., Huber-Lang M. (2020). Inflammation, thrombosis, and destruction: The three-headed cerberus of trauma- and SARS-CoV-2-induced ARDS. Front. Immunol..

[30] Bulla A., Hitze K.L. (1978). Acute respiratory infections: A review. Bull. World Health Organ..

[31] El-Hammadi M.M., Delgado A.V., Melguizo C., Prados J.C., Arias J.L. (2017). Folic acid-decorated and PEGylated PLGA nanoparticles for improving the antitumour activity of 5-fluorouracil. Int. J. Pharm..

[32] Mohammed H.A., Sulaiman G.M., Anwar S.S., Tawfeeq A.T., Khan R.A., Mohammed S.A.A., Al-Omar M.S., Alsharidah M., Rugaie O.A., Al-Amiery A.A. (2021). Quercetin against MCF7 and CAL51 breast cancer cell lines: Apoptosis, gene expression and cytotoxicity of nano-quercetin. Nanomedicine.

[33] Otaka E., Ooi T., Suzuki K. (1989). Examination of protein sequence homologies. VI. The evolution of Escherichia coli L7/L12 equivalent ribosomal proteins (‘A’ proteins), and the tertiary structure. Protein Seq. Data Anal..

[34] Hill M., Cunningham R.N., Hathout R.M., Johnston C., Hardy J.G., Migaud M.E. (2019). Formulation of antimicrobial tobramycin loaded PLGA nanoparticles via complexation with AOT. J. Funct. Biomater..

[35] Pandey P., Rahman M., Bhatt P.C., Beg S., Paul B., Al-Abbasi F.A., Hafeez A., Nadeem M.S., Baothman O., Anwar F. (2018). Implication of nano-antioxidant therapy for treatment of hepatocellular carcinoma using PLGA nanoparticles of rutin. Nanomedicine.

[36] Abdelkader D.H., Abosalha A.K., Khattab M.A., Aldosari B.N., Almurshedi A.S. (2021). A novel sustained anti-inflammatory effect of atorvastatin-calcium PLGA nanoparticles: In vitro optimization and in vivo evaluation. Pharmaceutics.

[37] Boltnarova B., Kubackova J., Skoda J., Stefela A., Smekalova M., Svacinova P., Pavkova I., Dittrich M., Scherman D., Zbytovska J. (2021). PLGA based nanospheres as a potent macrophage-specific drug delivery system. Nanomaterials.

[38] Wang Y., Li P., Tran T.T., Zhang J., Kong L. (2016). Manufacturing techniques and surface engineering of polymer based nanoparticles for targeted drug delivery to cancer. Nanomaterials.

[39] Astete C.E., Sabliov C.M. (2006). Synthesis and characterization of PLGA nanoparticles. J. Biomater. Sci. Polym. Ed..

[40] Makadia H.K., Siegel S.J. (2011). Poly lactic-co-glycolic acid (PLGA) as biodegradable controlled drug delivery carrier. Polymers.

[41] Garbayo E., Ansorena E., Blanco-Prieto M.J. (2012). Brain drug delivery systems for neurodegenerative disorders. Curr. Pharm. Biotechnol..

[42] Fernandez M., Barcia E., Fernández-Carballido A., Garcia L., Slowing K., Negro S. (2012). Controlled release of rasagiline mesylate promotes neuroprotection in a rotenone-induced advanced model of Parkinson’s disease. Int. J. Pharm..

[43] Formiga F.R., Pelacho B., Garbayo E., Abizanda G., Gavira J.J., Simon-Yarza T., Mazo M., Tamayo E., Jauquicoa C., Ortiz-de-Solorzano C. (2010). Sustained release of VEGF through PLGA microparticles improves vasculogenesis and tissue remodeling in an acute myocardial ischemia-reperfusion model. J. Control Release.

[44] Ospina-Villa J.D., Gómez-Hoyos C., Zuluaga-Gallego R., Triana-Chávez O. (2019). Encapsulation of proteins from Leishmania panamensis into PLGA particles by a single emulsion-solvent evaporation method. J. Microbiol. Methods.

[45] Zhang X., Intra J., Salem A.K. (2008). Comparative study of poly (lactic-co-glycolic acid)-poly ethyleneimine-plasmid DNA microparticles prepared using double emulsion methods. J. Microencapsul..

[46] Azizi M., Farahmandghavi F., Joghataei M.T., Zandi M., Imani M., Bakhtiari M., Omidian H. (2020). ChABC-loaded PLGA nanoparticles: A comprehensive study on biocompatibility, functional recovery, and axonal regeneration in animal model of spinal cord injury. Int. J. Pharm..

[47] Davoudi Z., Peroutka-Bigus N., Bellaire B., Wannemuehler M., Barrett T.A., Narasimhan B., Wang Q. (2018). Intestinal organoids containing poly(lactic-co-glycolic acid) nanoparticles for the treatment of inflammatory bowel diseases. J. Biomed. Mater. Res. A..

[48] Lee P.W., Pokorski J.K. (2018). Poly(lactic-co-glycolic acid) devices: Production and applications for sustained protein delivery. Wiley Interdiscip. Rev. Nanomed. Nanobiotechnol..

[49] Wan F., Yang M. (2016). Design of PLGA-based depot delivery systems for biopharmaceuticals prepared by spray drying. Int. J. Pharm..

[50] Nie H., Lee L.Y., Tong H., Wang C. (2008). PLGA/chitosan composites from a combination of spray drying and supercritical fluid foaming techniques: New carriers for DNA delivery. J. Control Release.

[51] Ding D., Zhu Q. (2018). Recent advances of PLGA micro/nanoparticles for the delivery of biomacromolecular therapeutics. Mater. Sci. Eng. C Mater. Biol. Appl..

[52] Liu J., Meisner D., Kwong E., Wu X.Y., Johnston M.R. (2007). A novel trans-lymphatic drug delivery system: Implantable gelatin sponge impregnated with PLGA-paclitaxel microspheres. Biomaterials.

[53] Wolburg H., Neuhaus J., Kniesel U., Krauss B., Schmid E.M., Ocalan M., Farrell C., Risau W. (1994). Modulation of tight junction structure in blood-brain barrier endothelial cells. Effects of tissue culture, second messengers and cocultured astrocytes. J. Cell Sci..

[54] Spindler L.M., Feuerhake A., Ladel S., Günday C., Flamm J., Günday-Türeli N., Türeli E., Tovar G.E.M., Schindowski K., Gruber-Traub C. (2021). Nano-in-micro-particles consisting of PLGA nanoparticles embedded in chitosan microparticles via spray-drying enhances their uptake in the olfactory mucosa. Front. Pharmacol..

[55] El-Maghawry E., Tadros M.I., Elkheshen S.A., Abd-Elbary A. (2020). Eudragit((R))-S100 coated PLGA nanoparticles for colon targeting of etoricoxib: Optimization and pharmacokinetic assessments in healthy human volunteers. Int. J. Nanomed..

[56] Yan X., Bernard J., Ganachaud F. (2021). Nanoprecipitation as a simple and straightforward process to create complex polymeric colloidal morphologies. Adv. Colloid Interface Sci..

[57] Lepeltier E., Bourgaux C., Couvreur P. (2014). Nanoprecipitation and the “Ouzo effect”: Application to drug delivery devices. Adv. Drug Deliv. Rev..

[58] Govender T., Stolnik S., Garnett M.C., Illum L., Davis S.S. (1999). PLGA nanoparticles prepared by nanoprecipitation: Drug loading and release studies of a water soluble drug. J. Control Release.

[59] Al-Humaidi R.B., Fayed B., Shakartalla S.B., Jagal J., Jayakumar M.N., Shareef Z.M.A., Sharif S.I., Noreddin A., Semreen M.H., Omar H.A. (2022). Optimum inhibition of MCF-7 breast cancer cells by efficient targeting of the macropinocytosis using optimized paclitaxel-loaded nanoparticles. Life Sci..

[60] Patra A., Satpathy S., Naik P.K., Kazi M., Hussain M.D. (2022). Folate receptor-targeted PLGA-PEG nanoparticles for enhancing the activity of genistein in ovarian cancer. Artif. Cells Nanomed. Biotechnol..

[61] Wang R., Bao Q., Clark A.G., Wang Y., Zhang S., Burgess D.J. (2022). Characterization and in vitro release of minocycline hydrochloride microspheres prepared via coacervation. Int. J. Pharm..

[62] Butreddy A., Gaddam R.P., Kommineni N., Dudhipala N., Voshavar C. (2021). PLGA/PLA-based long-acting injectable depot microspheres in clinical use: Production and characterization overview for protein/peptide delivery. Int. J. Mol. Sci..

[63] Park P.I.P., Makoid M., Jonnalagadda S. (2011). The design of flexible ciprofloxacin-loaded PLGA implants using a reversed phase separation/coacervation method. Eur. J. Pharm. Biopharm..

[64] Mu L., Feng S.S. (2001). Fabrication, characterization and in vitro release of paclitaxel (Taxol) loaded poly (lactic-co-glycolic acid) microspheres prepared by spray drying technique with lipid/cholesterol emulsifiers. J. Control Release.

[65] Gavini E., Chetoni P., Cossu M., Alvarez M.G., Saettone M.F., Giunchedi P. (2004). PLGA microspheres for the ocular delivery of a peptide drug, vancomycin using emulsification/spray-drying as the preparation method: In vitro/in vivo studies. Eur. J. Pharm. Biopharm..

[66] Sastre R.L., Olmo R., Teijon C., Muniz E., Teijon J.M., Blanco M.D. (2007). 5-Fluorouracil plasma levels and biodegradation of subcutaneously injected drug-loaded microspheres prepared by spray-drying poly(D,L-lactide) and poly(D,L-lactide-co-glycolide) polymers. Int. J. Pharm..

[67] Almoustafa H.A., Alshawsh M.A., Chik Z. (2017). Technical aspects of preparing PEG-PLGA nanoparticles as carrier for chemotherapeutic agents by nanoprecipitation method. Int. J. Pharm..

[68] Bodmeier R., Chen H., Tyle P., Jarosz P. (1991). Spontaneous formation of drug-containing acrylic nanoparticles. J. Microencapsul..

[69] Graham P.D., Brodbeck K.J., McHugh A.J. (1999). Phase inversion dynamics of PLGA solutions related to drug delivery. J. Control Release.

[70] Thomasin C., Ho N.T., Merkle H.P., Gander B. (1998). Drug microencapsulation by PLA/PLGA coacervation in the light of thermodynamics. 1. Overview and theoretical considerations. J. Pharm. Sci..

[71] Edelman R., Russell R.G., Losonsky G., Tall B.D., Tacket C.O., Levine M.M., Lewis D.H. (1993). Immunization of rabbits with enterotoxigenic E. coli colonization factor antigen (CFA/I) encapsulated in biodegradable microspheres of poly (lactide-co-glycolide). Vaccine.

[72] Kotta S., Aldawsari H.M., Badr-Eldin S.M., Binmahfouz L.S., Bakhaidar R.B., Sreeharsha N., Nair A.B., Ramnarayanan C. (2021). Lung targeted lipopolymeric microspheres of dexamethasone for the treatment of ARDS. Pharmaceutics.

[73] Almukainzi M., El-Masry T., Negm W., Elekhnawy E., Saleh A., Sayed A., Khattab M., Abdelkader D. (2022). Gentiopicroside PLGA Nanospheres: Fabrication, in vitro Characterization, Antimicrobial Action, and in vivo Effect for Enhancing Wound Healing in Diabetic Rats. Int. J. Nanomed..

[74] Aragon-Navas A., Rodrigo M.J., Garcia-Herranz D., Martinez T., Subias M., Mendez S., Ruberte J., Pampalona J., Bravo-Osuna I., Garcia-Feijoo J. (2022). Mimicking chronic glaucoma over 6 months with a single intracameral injection of dexamethasone/fibronectin-loaded PLGA microspheres. Drug Deliv..

[75] Zheng D., Wan C., Yang H., Xu L., Dong Q., Du C., Du J., Li F. (2020). Her2-Targeted Multifunctional Nano-Theranostic Platform Mediates Tumor Microenvironment Remodeling and Immune Activation for Breast Cancer Treatment. Int. J. Nanomed..

[76] Zhu K., Yao Y., Wang K., Shao F., Zhu Z., Song Y., Zhou Z., Jiang D., Lan X., Qin C. (2023). Berberin sustained-release nanoparticles were enriched in infarcted rat myocardium and resolved inflammation. J. Nanobiotechnol..

[77] Cappiello F., Casciaro B., Loffredo M.R., Puglisi E., Lin Q., Yang D., Conte G., d’Angelo I., Ungaro F., Ferrera L. (2022). Pulmonary safety profile of esc peptides and esc-peptide-loaded poly(lactide-co-glycolide) nanoparticles: A promising therapeutic approach for local treatment of lung infectious diseases. Pharmaceutics.

[78] Casciaro B., D’Angelo I., Zhang X., Loffredo M.R., Conte G., Cappiello F., Quaglia F., Di Y.-P.P., Ungaro F., Mangoni M.L. (2019). Poly(lactide-co-glycolide) nanoparticles for prolonged therapeutic efficacy of esculentin-1a-derived antimicrobial peptides against *Pseudomonas aeruginosa* lung infection: In vitro and in vivo studies. Biomacromolecules.

[79] Qu S., Zhao L., Zhu J., Wang C., Dai C., Guo H., Hao Z. (2017). Preparation and testing of cefquinome-loaded poly lactic-co-glycolic acid microspheres for lung targeting. Drug Deliv..

[80] SreeHarsha N., Venugopala K.N., Nair A.B., Roopashree T.S., Attimarad M., Hiremath J.G., Al-Dhubiab B., Ramnarayanan C., Shinu P., Handral M. (2019). An efficient, lung-targeted, drug-delivery system to treat asthma via microparticles. Drug Des. Dev. Ther..

[81] Hoyle G.W., Chen J., Schlueter C.F., Mo Y., Humphrey D.M., Rawson G., Nino J.A., Carson K.H. (2016). Development and assessment of countermeasure formulations for treatment of lung injury induced by chlorine inhalation. Toxicol. Appl. Pharmacol..

[82] Nassar T., Rohald A., Naraykin N., Barasch D., Amsalem O., Prabhu P., Kotler M., Benita S. (2019). Nanocapsules embedded in microparticles for enhanced oral bioavailability and efficacy of Lopinavir as an anti-AIDS drug. J. Drug Target.

[83] Yang X., Wu S., Xie W., Cheng A., Yang L., Hou Z., Jin X. (2017). Dual-drug loaded nanoneedles with targeting property for efficient cancer therapy. J. Nanobiotechnol..

[84] Maleki H., Hosseini Najafabadi M.R., Webster T.J., Hadjighassem M.R., Sadroddiny E., Ghanbari H., Khosravani M., Adabi M. (2021). Effect of Paclitaxel/etoposide co-loaded polymeric nanoparticles on tumor size and survival rate in a rat model of glioblastoma. Int. J. Pharm..

[85] Bairagi U., Mittal P., Singh J., Mishra B. (2018). Preparation, characterization, and in vivo evaluation of nano formulations of ferulic acid in diabetic wound healing. Drug Dev. Ind. Pharm..

[86] Katara R., Sachdeva S., Majumdar D.K. (2017). Enhancement of ocular efficacy of aceclofenac using biodegradable PLGA nanoparticles: Formulation and characterization. Drug Deliv. Transl. Res..

[87] Ma X., Kuang L., Yin Y., Tang L., Zhang Y., Fan Q., Wang B., Dong Z., Wang W., Yin T. (2023). Tumor-antigen activated dendritic cell membrane-coated biomimetic nanoparticles with orchestrating immune responses promote therapeutic efficacy against glioma. ACS Nano.

[88] Oh Y.J., Lee J., Seo J.Y., Rhim T., Kim S.H., Yoon H.J., Lee K.Y. (2011). Preparation of budesonide-loaded porous PLGA microparticles and their therapeutic efficacy in a murine asthma model. J. Control Release.

[89] Pálinkó-Biró E., Rónaszèki G., Merkle H.P., Gander B. (2001). Release kinetics and immunogenicity of parvovirus microencapsulated in PLA/PLGA microspheres. Int. J. Pharm..

[90] Johansen P., Moon L., Tamber H., Merkle H.P., Gander B., Sesardic D. (1999). Immunogenicity of single-dose diphtheria vaccines based on PLA/PLGA microspheres in guinea pigs. Vaccine.

[91] Kolge H., Patil G., Jadhav S., Ghormade V. (2023). A pH-tuned chitosan-PLGA nanocarrier for fluconazole delivery reduces toxicity and improves efficacy against resistant Candida. Int. J. Biol. Macromol..

[92] Fernández-Álvarez F., Caro C., García-García G., García-Martín M.L., Arias J.L. (2021). Engineering of stealth (maghemite/PLGA)/chitosan (core/shell)/shell nanocomposites with potential applications for combined MRI and hyperthermia against cancer. J. Mater. Chem. B.

[93] Meena R., Kumar S., Kumar R., Gaharwar U.S., Rajamani P. (2017). PLGA-CTAB curcumin nanoparticles: Fabrication, characterization and molecular basis of anticancer activity in triple negative breast cancer cell lines (MDA-MB-231 cells). Biomed. Pharmacother..

[94] Kang B.-S., Choi J.-S., Lee S.-E., Lee J.-K., Kim T.-H., Jang W.S., Tunsirikongkon A., Kim J.-K., Park J.-S. (2017). Enhancing the in vitro anticancer activity of albendazole incorporated into chitosan-coated PLGA nanoparticles. Carbohydr. Polym..

[95] Pawar D., Mangal S., Goswami R., Jaganathan K.S. (2013). Development and characterization of surface modified PLGA nanoparticles for nasal vaccine delivery: Effect of mucoadhesive coating on antigen uptake and immune adjuvant activity. Eur. J. Pharm. Biopharm..

[96] Ibrahim M., Ramadan E., Elsadek N.E., Emam S.E., Shimizu T., Ando H., Ishima Y., Elgarhy O.H., Sarhan H.A., Hussein A.K. (2022). Polyethylene glycol (PEG): The nature, immunogenicity, and role in the hypersensitivity of PEGylated products. J. Control. Release.

[97] Vllasaliu D., Fowler R., Stolnik S. (2014). PEGylated nanomedicines: Recent progress and remaining concerns. Expert Opin. Drug Deliv..

[98] Owens D.E., Peppas N.A. (2006). Opsonization, biodistribution, and pharmacokinetics of polymeric nanoparticles. Int. J. Pharm..

[99] Amoozgar Z., Park J., Lin Q., Yeo Y. (2012). Low Molecular-Weight Chitosan as a pH-Sensitive Stealth Coating for Tumor-Specific Drug Delivery. Mol. Pharm..

[100] Majumder J., Minko T. (2021). Targeted Nanotherapeutics for Respiratory Diseases: Cancer, Fibrosis, and Coronavirus. Adv. Ther..

[101] Koerner J., Horvath D., Groettrup M. (2019). Harnessing Dendritic Cells for Poly (D,L-lactide-co-glycolide) Microspheres (PLGA MS)—Mediated Anti-tumor Therapy. Front. Immunol..

[102] Yang Y., Ding Y., Fan B., Wang Y., Mao Z., Wang W., Wu J. (2020). Inflammation-targeting polymeric nanoparticles deliver sparfloxacin and tacrolimus for combating acute lung sepsis. J. Control. Release.

[103] Zhang C.Y., Lin W., Gao J., Shi X., Davaritouchaee M., Nielsen A.E., Mancini R.J., Wang Z. (2019). pH-Responsive Nanoparticles Targeted to Lungs for Improved Therapy of Acute Lung Inflammation/Injury. ACS Appl. Mater. Interfaces.

[104] Spence S., Greene M.K., Fay F., Hams E., Saunders S.P., Hamid U., Fitzgerald M., Beck J., Bains B.K., Smyth P. (2015). Targeting Siglecs with a sialic acid–decorated nanoparticle abrogates inflammation. Sci. Transl. Med..

[105] Prakash Y.S., Halayko A.J., Gosens R., Panettieri R.A., Camoretti-Mercado B., Penn R.B., ATS Assembly on Respiratory Structure and Function (2017). An official american thoracic society research statement: Current challenges facing research and therapeutic advances in airway remodeling. Am. J. Respir. Crit Care Med..

[106] Stern J., Pier J., Litonjua A.A. (2020). Asthma epidemiology and risk factors. Semin. Immunopathol..

[107] Zhao Y., Wang Y., Ran F., Cui Y., Liu C., Zhao Q., Gao Y., Wang D., Wang S. (2017). A comparison between sphere and rod nanoparticles regarding their in vivo biological behavior and pharmacokinetics. Sci. Rep..

[108] Lee S.-Y., Ferrari M., Decuzzi P. (2009). Shaping nano-/micro-particles for enhanced vascular interaction in laminar flows. Nanotechnology.

[109] Decuzzi P., Godin B., Tanaka T., Lee S.-Y., Chiappini C., Liu X., Ferrari M. (2010). Size and shape effects in the biodistribution of intravascularly injected particles. J. Control. Release.

[110] Park J.Y., Chu G.E., Park S., Park C., Aryal S., Kang W.J., Gil Cho W., Key J. (2020). Therapeutic Efficacy of Curcumin Enhanced by Microscale Discoidal Polymeric Particles in a Murine Asthma Model. Pharmaceutics.

[111] Patel B., Rashid J., Ahsan F. (2017). Aerosolizable modified-release particles of montelukast improve retention and availability of the drug in the lungs. Eur. J. Pharm. Sci..

[112] Perry J.L., Reuter K.G., Kai M.P., Herlihy K.P., Jones S.W., Luft J.C., Napier M., Bear J.E., DeSimone J.M. (2012). PEGylated PRINT Nanoparticles: The Impact of PEG Density on Protein Binding, Macrophage Association, Biodistribution, and Pharmacokinetics. Nano Lett..

[113] Li J., Zheng H., Xu E.-Y., Moehwald M., Chen L., Zhang X., Mao S. (2021). Inhalable PLGA microspheres: Tunable lung retention and systemic exposure via polyethylene glycol modification. Acta Biomater..

[114] Akdis M., Akdis C.A. (2009). Therapeutic manipulation of immune tolerance in allergic disease. Nat. Rev. Drug Discov..

[115] Akdis C.A. (2012). Therapies for allergic inflammation: Refining strategies to induce tolerance. Nat. Med..

[116] Ma A., Malynn B.A. (2012). A20: Linking a complex regulator of ubiquitylation to immunity and human disease. Nat. Rev. Immunol..

[117] Luo X.-Q., Zhong J.-W., Qiu S.-Y., Zhi M., Yang L.-Q., Zhou Y.-L., Zhou F.-X., Yang P.-C., Liu D.-B., Mo L.-H. (2020). A20-OVA Nanoparticles Inhibit Allergic Asthma in a Murine Model. Inflammation.

[118] Li X., Zhang Y., Pei W., Zhang M., Yang H., Zhong M., Kong X., Xu Y., Zhu X., Chen T. (2020). LncRNA *Dnmt3aos* regulates *Dnmt3a* expression leading to aberrant DNA methylation in macrophage polarization. FASEB J..

[119] Pei W., Li X., Bi R., Zhang X., Zhong M., Yang H., Zhang Y., Lv K. (2021). Exosome membrane-modified M2 macrophages targeted nanomedicine: Treatment for allergic asthma. J. Control. Release.

[120] GBD 2017 Disease and Injury Incidence and Prevalence Collaborators (2018). Global, regional, and national incidence, prevalence, and years lived with disability for 354 diseases and injuries for 195 countries and territories, 1990–2017: A systematic analysis for the Global Burden of Disease Study 2017. Lancet.

[121] Matera M.G., Page C.P., Cazzola M. (2011). Novel bronchodilators for the treatment of chronic obstructive pulmonary disease. Trends Pharmacol. Sci..

[122] Barnes P.J. (2010). Inhaled Corticosteroids in COPD: A Controversy. Respiration.

[123] Gordon S., Taylor P.R. (2005). Monocyte and macrophage heterogeneity. Nat. Rev. Immunol..

[124] Martinez F.O., Gordon S., Locati M., Mantovani A. (2006). Transcriptional Profiling of the Human Monocyte-to-Macrophage Differentiation and Polarization: New Molecules and Patterns of Gene Expression. J. Immunol..

[125] Mosser D.M., Edwards J.P. (2008). Exploring the full spectrum of macrophage activation. Nat. Rev. Immunol..

[126] van Noort J.M., Bsibsi M., Nacken P.J., Gerritsen W.H., Amor S., Holtman I.R., Boddeke E., van Ark I., Leusink-Muis T., Folkerts G. (2013). Activation of an immune-regulatory macrophage response and inhibition of lung inflammation in a mouse model of COPD using heat-shock protein alpha B-crystallin-loaded PLGA microparticles. Biomaterials.

[127] Fire A., Xu S., Montgomery M.K., Kostas S.A., Driver S.E., Mello C.C. (1998). Potent and specific genetic interference by double-stranded RNA in Caenorhabditis elegans. Nature.

[128] Fujita Y., Takeshita F., Kuwano K., Ochiya T. (2013). RNAi Therapeutic Platforms for Lung Diseases. Pharmaceuticals.

[129] Kishore A., Borucka J., Petrkova J., Petrek M. (2014). Novel Insights into miRNA in Lung and Heart Inflammatory Diseases. Mediat. Inflamm..

[130] Ebrahimi A., Sadroddiny E. (2015). MicroRNAs in lung diseases: Recent findings and their pathophysiological implications. Pulm. Pharmacol. Ther..

[131] Sato T., Liu X., Nelson A., Nakanishi M., Kanaji N., Wang X., Kim M., Li Y., Sun J., Michalski J. (2010). Reduced miR-146a Increases Prostaglandin E_2_ in Chronic Obstructive Pulmonary Disease Fibroblasts. Am. J. Respir. Crit. Care Med..

[132] Saxena J., Bisen M., Misra A., Srivastava V.K., Kaushik S., Siddiqui A.J., Mishra N., Singh A., Jyoti A. (2022). Targeting COPD with PLGA-Based Nanoparticles: Current Status and Prospects. BioMed Res. Int..

[133] Yin H., Kanasty R.L., Eltoukhy A.A., Vegas A.J., Dorkin J.R., Anderson D.G. (2014). Non-viral vectors for gene-based therapy. Nat. Rev. Genet..

[134] Mohamed A., Pekoz A.Y., Ross K., Hutcheon G.A., Saleem I.Y. (2019). Pulmonary delivery of Nanocomposite Microparticles (NCMPs) incorporating miR-146a for treatment of COPD. Int. J. Pharm..

[135] Vij N., Min T., Bodas M., Gorde A., Roy I. (2016). Neutrophil targeted nano-drug delivery system for chronic obstructive lung diseases. Nanomedicine.

[136] Endres T.M., Konstan M.W. (2022). What Is Cystic Fibrosis?. J. Am. Med. Assoc..

[137] Ooi C.Y., Durie P.R. (2016). Cystic fibrosis from the gastroenterologist’s perspective. Nat. Rev. Gastroenterol. Hepatol..

[138] Elborn J.S. (2016). Cystic fibrosis. Lancet.

[139] Ratjen F., Bell S.C., Rowe S.M., Goss C.H., Quittner A.L., Bush A. (2015). Cystic fibrosis. Nat. Rev. Dis. Prim..

[140] Heijerman H. (2005). Infection and inflammation in cystic fibrosis: A short review. J. Cyst. Fibros..

[141] Høiby N. (2002). Understanding bacterial biofilms in patients with cystic fibrosis: Current and innovative approaches to potential therapies. J. Cyst. Fibros..

[142] Zhang L., Pornpattananangkul D., Hu C.-M., Huang C.-M. (2010). Development of Nanoparticles for Antimicrobial Drug Delivery. Curr. Med. Chem..

[143] Lakshminarayanan R., Ye E., Young D.J., Li Z., Loh X.J. (2018). Recent Advances in the Development of Antimicrobial Nanoparticles for Combating Resistant Pathogens. Adv. Healthc. Mater..

[144] Türeli N.G., Torge A., Juntke J., Schwarz B.C., Schneider-Daum N., Türeli A.E., Lehr C.-M., Schneider M. (2017). Ciprofloxacin-loaded PLGA nanoparticles against cystic fibrosis *P. aeruginosa* lung infections. Eur. J. Pharm. Biopharm..

[145] McEwan D.L., Kirienko N.V., Ausubel F.M. (2012). Host Translational Inhibition by *Pseudomonas aeruginosa* Exotoxin A Triggers an Immune Response in *Caenorhabditis elegans*. Cell Host Microbe.

[146] Weber A., Zimmermann C., Mausberg A.K., Dehmel T., Kieseier B.C., Hartung H., Hofstetter H.H. (2016). *Pseudomonas aeruginosa* and its bacterial components influence the cytokine response in thymocytes and splenocytes. Infect. Immun..

[147] Zanjani L.S., Shapouri R., Dezfulian M., Mahdavi M., Ardestani M.S. (2019). Exotoxin A-PLGA nanoconjugate vaccine against *Pseudomonas aeruginosa* infection: Protectivity in murine model. World J. Microbiol. Biotechnol..

[148] Kortright K.E., Chan B.K., Koff J.L., Turner P.E. (2019). Phage therapy: A renewed approach tocombat antibiotic-resistant bacteria. Cell Host Microbe.

[149] Donlan R.M. (2009). Preventing biofilms of clinically relevant organisms using bacteriophage. Trends Microbiol..

[150] Millette G., Langlois J., Brouillette E., Frost E.H., Cantin A.M., Malouin F. (2019). Despite antagonism in vitro, *Pseudomonas aeruginosa* enhances *Staphylococcus aureus* colonization in a murine lung infection model. Front. Microbiol..

[151] Kalelkar P.P., Moustafa D.A., Riddick M., Goldberg J.B., McCarty N.A., García A.J. (2022). Bacteriophage-loaded poly(lactic-co-glycolic acid) microparticles mitigate *Staphylococcus aureus* infection and cocultures of *Staphylococcus aureus* and *Pseudomonas aeruginosa*. Adv. Healthc. Mater..

[152] Dhooghe B., Noël S., Huaux F., Leal T. (2014). Lung inflammation in cystic fibrosis: Pathogenesis and novel therapies. Clin. Biochem..

[153] Duncan G.A., Jung J., Hanes J., Suk J.S. (2016). The mucus barrier to inhaled gene therapy. Mol. Ther..

[154] Ma J., Rubin B.K., Voynow J.A. (2018). Mucins, mucus, and goblet cells. Chest.

[155] Garcia-Diaz M., Birch D., Wan F., Nielsen H.M. (2018). The role of mucus as an invisible cloak to transepithelial drug delivery by nanoparticles. Adv. Drug Deliv. Rev..

[156] Wine J.J., Hansson G.C., König P., Joo N.S., Ermund A., Pieper M. (2018). Progress in understanding mucus abnormalities in cystic fibrosis airways. J. Cyst. Fibros..

[157] Bear C.E. (2013). 50 years ago in the Journal of Pediatrics: The effect of N-acetylcysteine on the viscosity of tracheobronchial secretions in cystic fibrosis of the pancreas. J. Pediatr..

[158] Cristallini C., Barbani N., Ventrelli L., Summa L., Filippi S., Capelôa T., Vitale E., Albera C., Messore B., Giachino C. (2019). Biodegradable microparticles designed to efficiently reach and act on cystic fibrosis mucus barrier. Mater. Sci. Eng. C Mater. Biol. Appl..

[159] Ware L.B., Matthay M.A. (2000). The acute respiratory distress syndrome. N. Engl. J. Med..

[160] Bellani G., Laffey J.G., Pham T., Fan E., Brochard L., Esteban A., Gattinoni L., Haren F.V., Larsson A., McAuley D.F. (2016). Epidemiology, patterns of care, and mortality for patients with acute respiratory distress syndrome in intensive care units in 50 countries. J. Am. Med. Assoc..

[161] Liu C., Xiao K., Xie L. (2022). Advances in the use of exosomes for the treatment of ALI/ARDS. Front. Immunol..

[162] Fan E., Brodie D., Slutsky A.S. (2018). Acute respiratory distress syndrome: Advances in diagnosis and treatment. J. Am. Med. Assoc..

[163] Villar J., Ferrando C., Martínez D., Ambrós A., Muñoz T., Soler J.A., Aguilar G., Alba F., González-Higueras E., Conesa L.A. (2020). Dexamethasone treatment for the acute respiratory distress syndrome: A multicentre, randomised controlled trial. Lancet Respir. Med..

[164] Waljee A.K., Rogers M.A.M., Lin P., Singal A.G., Stein J.D., Marks R.M., Ayanian J.Z., Nallamothu B.K. (2017). Short term use of oral corticosteroids and related harms among adults in the United States: Population based cohort study. Br. Med. J..

[165] Nourshargh S., Alon R. (2014). Leukocyte migration into inflamed tissues. Immunity.

[166] Park J.H., Jiang Y., Zhou J., Gong H., Mohapatra A., Heo J., Gao W., Fang R.H., Zhang L. (2021). Genetically engineered cell membrane-coated nanoparticles for targeted delivery of dexamethasone to inflamed lungs. Sci. Adv..

[167] Lang J.D., McArdle P.G., O’Reilly P.J., Matalon S. (2002). Oxidant-antioxidant balance in acute lung injury. Chest.

[168] Hsieh L.T., Frey H., Nastase M., Tredup C., Hoffmann A., Poluzzi C., Zeng-Brouwers J., Manon-Jensen T., Schröder K. (2016). Bimodal role of NADPH oxidases in the regulation of biglycan-triggered IL-1beta synthesis. Matrix Biol..

[169] Jin F., Liu D., Yu H., Qi J., You Y., Xu X., Kang X., Wang X., Lu K., Ying X. (2019). Sialic acid-functionalized PEG-PLGA microspheres loading mitochondrial-targeting-modified curcumin for acute lung injury therapy. Mol. Pharm..

[170] de Menezes B.R.C., Rodrigues K.F., Schatkoski V.M., Pereira R.M., Ribas R.G., Montanheiro T.L.D.A., Thim G.P. (2021). Current advances in drug delivery of nanoparticles for respiratory disease treatment. J. Mater. Chem. B.

[171] Piters W.A.A.D.S., Binkowska J., Bogaert D. (2020). Early life microbiota and respiratory tract infections. Cell Host Microbe.

[172] Tali S.H.S., LeBlanc J.J., Sadiq Z., Oyewunmi O.D., Camargo C., Nikpour B., Armanfard N., Sagan S.M., Jahanshahi-Anbuhi S. (2021). Tools and techniques for severe acute respiratory syndrome coronavirus 2 (SARS-CoV-2)/COVID-19 detection. Clin. Microbiol. Rev..

[173] Dhama K., Khan S., Tiwari R., Sircar S., Bhat S., Malik Y.S., Singh K.P., Chaicumpa W., Bonilla-Aldana D.K., Rodriguez-Morales A.J. (2020). Coronavirus disease 2019-COVID-19. Clin. Microbiol. Rev..

[174] Wiersinga W.J., Rhodes A., Cheng A.C., Peacock S.J., Prescott H.C. (2020). Pathophysiology, transmission, diagnosis, and treatment of coronavirus disease 2019 (COVID-19): A review. J. Am. Med. Assoc..

[175] Chauhan G., Madou M.J., Kalra S., Chopra V., Ghosh D., Martinez-Chapa S.O. (2020). Nanotechnology for COVID-19: Therapeutics and vaccine research. ACS Nano.

[176] Satija S., Mehta M., Sharma M., Prasher P., Gupta G., Chellappan D.K., Dua K. (2020). Vesicular drug delivery systems as theranostics in COVID-19. Future Med. Chem..

[177] Jin Y., Yang H., Ji W., Wu W., Chen S., Zhang W., Duan G. (2020). Virology, epidemiology, pathogenesis, and control of COVID-19. Viruses.

[178] Wang M., Cao R., Zhang L., Yang X., Liu J., Xu M., Shi Z., Hu Z., Zhong W., Xiao G. (2020). Remdesivir and chloroquine effectively inhibit the recently emerged novel coronavirus (2019-nCoV) in vitro. Cell Res..

[179] Taddio A., Ipp M., Thivakaran S., Jamal A., Parikh C., Smart S., Sovran J., Stephens D., Katz J. (2012). Survey of the prevalence of immunization non-compliance due to needle fears in children and adults. Vaccine.

[180] Patki M., Palekar S., Reznik S., Patel K. (2021). Self-injectable extended release formulation of Remdesivir (SelfExRem): A potential formulation alternative for COVID-19 treatment. Int. J. Pharm..

[181] Skevaki C., Fragkou P.C., Cheng C., Xie M., Renz H. (2020). Laboratory characteristics of patients infected with the novel SARS-CoV-2 virus. J. Infect..

[182] Tan Q., He L., Meng X., Wang W., Pan H., Yin W., Zhu T., Huang X., Shan H. (2021). Macrophage biomimetic nanocarriers for anti-inflammation and targeted antiviral treatment in COVID-19. J. Nanobiotechnol..

[183] Pena-Silva R., Duffull S.B., Steer A.C., Jaramillo-Rincon S.X., Gwee A., Zhu A. (2021). Pharmacokinetic considerations on the repurposing of ivermectin for treatment of COVID-19. Br. J. Clin. Pharmacol..

[184] Zheng J., Lu C., Ding Y., Zhang J., Tan F., Liu J., Yang G., Wang Y., Li Z., Yang M. (2022). Red blood cell-hitchhiking mediated pulmonary delivery of ivermectin: Effects of nanoparticle properties. Int. J. Pharm..

[185] Brenner J.S., Mitragotri S., Muzykantov V.R. (2021). Red blood cell hitchhiking: A novel approach for vascular delivery of nanocarriers. Annu. Rev. Biomed. Eng..

[186] Katz S.E., Williams D.J. (2018). Pediatric community-acquired pneumonia in the United States: Changing epidemiology, diagnostic and therapeutic challenges, and creas for future research. Infect. Dis. Clin. N. Am..

[187] Grousd J.A., Rich H.E., Alcorn J.F. (2019). Host-pathogen interactions in gram-positive bacterial pneumonia. Clin. Microbiol. Rev..

[188] Hageman J.C., Uyeki T.M., Francis J.S., Jernigan D.B., Wheeler J.G., Bridges C.B., Barenkamp S.J., Sievert D.M., Srinivasan A., Doherty M.C. (2006). Severe community-acquired pneumonia due to *Staphylococcus aureus*, 2003–2004 influenza season. Emerg. Infect. Dis..

[189] Diep B.A., Le V.T.M., Badiou C., Le H.N., Pinheiro M.G., Duong A.H., Wang X., Dip E.C., Aguiar-Alves F., Basuino L. (2016). IVIG-mediated protection against necrotizing pneumonia caused by MRSA. Sci. Transl. Med..

[190] Flemming H., Wingender J., Szewzyk U., Steinberg P., Rice S.A., Kjelleberg S. (2016). Biofilms: An emergent form of bacterial life. Nat. Rev. Microbiol..

[191] Lin X., He J., Li W., Qi Y., Hu H., Zhang D., Xu F., Chen X., Zhou M. (2021). Lung-targeting lysostaphin microspheres for methicillin-resistant *Staphylococcus aureus* pneumonia treatment and prevention. ACS Nano.

[192] Sievert D.M., Ricks P., Edwards J.R., Schneider A., Patel J., Srinivasan A., Kallen A., Limbago B., Fridkin S. (2013). National healthcare safety network (NHSN) team and participating NHSN Facilities. Antimicrobial-resistant pathogens associated with healthcare-associated infections: Summary of data reported to the National Healthcare Safety Network at the Centers for Disease Control and Prevention, 2009–2010. Infect. Control Hosp. Epidemiol..

[193] Brenwald N.P., Jevons G., Andrews J.M., Xiong J., Hawkey P.M., Wise R. (2003). An outbreak of a CTX-M-type beta-lactamase-producing *Klebsiella* pneumoniae: The importance of using cefpodoxime to detect extended-spectrum beta-lactamases. J. Antimicrob. Chemother..

[194] Schaefers M.M., Duan B., Mizrahi B., Lu R., Reznor G., Kohane D.S., Priebe G.P. (2018). PLGA-encapsulation of the *Pseudomonas aeruginosa* PopB vaccine antigen improves Th17 responses and confers protection against experimental acute pneumonia. Vaccine.

[195] Qu S., Dai C., Yang F., Huang T., Hao Z., Tang Q., Wang H., Zhang Y. (2019). Cefquinome-loaded microsphere formulations in protection against pneumonia with *Klebsiella* pneumonia Infection and inflammatory response in rats. Pharm. Res..

[196] Mogharabi M., Abdollahi M., Faramarzi M.A. (2014). Toxicity of nanomaterials; an undermined issue. DARU J. Pharm. Sci..

[197] Provenzano F., Nyberg S., Giunti D., Torazza C., Parodi B., Bonifacino T., Usai C., Rosbo N.K.D., Milanese M., Uccelli A. (2022). Micro-RNAs shuttled by extracellular vesicles secreted from mesenchymal stem cells dampen astrocyte pathological activation and support neuroprotection in in-vitro models of ALS. Cells.

[198] Zhu S., Xing H., Gordiichuk P., Park J., Mirkin C.A. (2018). PLGA spherical nucleic acids. Adv. Mater..

[199] Ma S., Wang C., Dong Y., Jing W., Wei P., Peng C., Liu Z., Zhao B., Wang Y. (2022). Microsphere-gel composite system with mesenchymal stem cell recruitment, antibacterial, and immunomodulatory properties promote bone regeneration via sequential release of LL37 and W9 peptides. ACS Appl. Mater. Interfaces.

[200] Bittner B., Ronneberger B., Zange R., Volland C., Anderson J.M., Kissel T. (1998). Bovine serum albumin loaded poly(lactide-co-glycolide) microspheres: The influence of polymer purity on particle characteristics. J. Microencapsul..

[201] Johansen P., Men Y., Audran R., Corradin G., Merkle H.P., Gander B. (1998). Improving stability and release kinetics of microencapsulated tetanus toxoid by co-encapsulation of additives. Pharm. Res..

[202] Kuzmov A., Minko T. (2015). Nanotechnology approaches for inhalation treatment of lung diseases. J. Control Release.

[203] Bonner J.C. (2010). Nanoparticles as a potential cause of pleural and interstitial lung disease. Proc. Am. Thorac. Soc..

[204] Shi J., Kantoff P.W., Wooster R., Farokhzad O.C. (2017). Cancer nanomedicine: Progress, challenges and opportunities. Nat. Rev. Cancer.

[205] Hare J.I., Lammers T., Ashford M.B., Puri S., Storm G., Barry S.T. (2017). Challenges and strategies in anti-cancer nanomedicine development: An industry perspective. Adv. Drug Deliv. Rev..

[206] Jiang H.L., Jin J.F., Hu Y.Q., Zhu K.J. (2004). Improvement of protein loading and modulation of protein release from poly(lactide-co-glycolide) microspheres by complexation of proteins with polyanions. J. Microencapsul..

[207] Suk J.S. (2016). Could recent advances in DNA-loaded nanoparticles lead to effective inhaled gene therapies?. Nanomedicine.

[208] Lee H., Jeong S.W., Jung E., Lee D. (2020). Dexamethasone-loaded H_2_O_2_-activatable anti-inflammatory nanoparticles for on-demand therapy of inflammatory respiratory diseases. Nanomedicine.

[209] Hossen S., Hossain M.K., Basher M.K., Mia M.N.H., Rahman M.T., Uddin M.J. (2019). Smart nanocarrier-based drug delivery systems for cancer therapy and toxicity studies: A review. J. Adv. Res..

[210] Wilhelm S., Tavares A.J., Dai Q., Ohta S., Audet J., Dvorak H.F., Chan W.C.W. (2016). Analysis of nanoparticle delivery to tumours. Nat. Rev. Mater..

[211] Genchi G.G., Marino A., Tapeinos C., Ciofani G. (2017). Smart materials meet multifunctional biomedical devices: Current and prospective implications for nanomedicine. Front. Bioeng. Biotechnol..

